# Expectation-Maximization-Maximization: A Feasible MLE Algorithm for the Three-Parameter Logistic Model Based on a Mixture Modeling Reformulation

**DOI:** 10.3389/fpsyg.2017.02302

**Published:** 2018-01-05

**Authors:** Chanjin Zheng, Xiangbin Meng, Shaoyang Guo, Zhengguang Liu

**Affiliations:** ^1^School of Psychology, Jiangxi Normal University, Nanchang, China; ^2^Faculty of Education, Northeast Normal University, Changchun, China; ^3^Key Laboratory of Applied Statistics of Moe, Northeast Normal University, Changchun, China; ^4^National Cooperative Innovation Center for Assessment and Improvement of Basic Education Quality, Beijing Normal University, Beijing, China

**Keywords:** EMM, Bayesian EM, MLE, mixture modeling, 3PL

## Abstract

Stable maximum likelihood estimation (MLE) of item parameters in 3PLM with a modest sample size remains a challenge. The current study presents a mixture-modeling approach to 3PLM based on which a feasible Expectation-Maximization-Maximization (EMM) MLE algorithm is proposed. The simulation study indicates that EMM is comparable to the Bayesian EM in terms of bias and RMSE. EMM also produces smaller standard errors (SEs) than MMLE/EM. In order to further demonstrate the feasibility, the method has also been applied to two real-world data sets. The point estimates in EMM are close to those from the commercial programs, BILOG-MG and flexMIRT, but the SEs are smaller.

## 1. Introduction

In the field of educational measurement, item response theory (IRT) models are a powerful tool aimed at providing an appropriate representation of students' test-taking behavior, and produce accurate estimates of students' ability. IRT models are expected to capture the underlying response processes such as students' ability and other strategies students might take. One of the most common strategies is guessing behavior when students cannot solve a problem correctly. The guessing strategy is prevalent particularly for multiple-choice questions in a low-stakes test (Lord, 1980; Baker and Kim, 2004; Cao and Stokes, 2008; Woods, 2008). To count for guessing, the three-parameter logistic model (3PLM; Birnbaum, 1968) has been commonly used in many applications of IRT in the measurement industry.

Despite of its popularity, however, several studies have pointed out technical and theoretical issues regarding the *c*-parameter (the guessing parameter) and its interpretation (Lord, 1974, 1980; Kolen, 1981; Thissen and Wainer, 1982; Holland, 1990; Hambleton et al., 1991; Yen et al., 1991; San Martín et al., 2006, 2013, 2015; Woods, 2008; Maris and Bechger, 2009; McDonald, 2013). The current paper focuses on one of long-standing issues for the 3PLM, the item parameter estimation, which has proved to be challenging (Thissen and Wainer, 1982). Mislevy (1986) pointed out that the essential difficulty is the sparse data for the guessing parameter, yielding unstable maximum likelihood estimates (MLEs). The well-established marginal maximum likelihood estimation via expectation-maximization (MMLE/EM) algorithm (Bock and Aitkin, 1981) is not feasible in this case. According to Thissen and Wainer (1982), the sample size required for obtaining MLE for 3PLM with acceptable standard errors using MMLE/EM is about 10,000 and, for some items, as large as 1,000,000 which seems prohibitively expensive or impractical even now. A most recent empirical study testifying to this claim is Tay et al. (2016) in which the researchers recommended that a sample of 20,000 is desirable when fitting a 3PLM with covariate (Tay et al., 2013). In fact, Thissen and Wainer (1982) even concluded that “naked maximum likelihood estimation for the three-parameter model is not a technique that is likely to give happy results.” San Martín et al. (2015) showed that the 3PLM is not even identifiable if a fixed-effects approach is adopted and the question of identifiability of the random-effects 3PLM is still open.

By now only some decent Bayesian methods have been developed, such as Bayesian EM (Mislevy, 1986), Bayesian joint estimation (Swaminathan and Gifford, 1986), and MCMC (Patz and Junker, 1999) MLE, however, enjoys favorable statistical properties (Casella and Berger, 2002). It may avoid some issues in Bayesian methods, such as assigning priors, convergence checking in MCMC, etc. and, thus, usually is preferred for parameter estimation. It would be a valuable addition to existing methods if that a practical feasible MLE algorithm could be developed for the 3PLM. The current study intends to make some contribution in this respect. The authors propose a feasible EM algorithm for the 3PLM, namely expectation-maximization-maximization (EMM). EMM can be considered as a modified version of the MMLE/EM algorithm (Bock and Aitkin, 1981) and the extra maximization step is especially designed for the guessing parameter due to a different setup for the complete data based on a mixture-modeling reformulation of the 3PLM.

This mixture-modeling approach to the 3PLM is not entirely new in the IRT literature. Mixture modeling is a well-established tradition in IRT, especially for Rasch models (von Davier and Rost, 2006). As for the 3PLM in particular, Hutchinson (1991) first discussed the two underlying processes of guessing and answering based on ability which points to the idea of mixture modeling for the 3PLM. San Martín et al. (2006) proposed an ability-based guessing model to describe the interaction between guessing behavior and examinee's ability based on this perspective. von Davier (2009) further presented two different interpretations of the 3PLM from this standpoint. One may even easily notice that the reformulation of the 3PLM developed by the current study can be considered as a special case of the HTBRID model (Yamamoto, 1982) and the general diagnostic model (von Davier, 2008), both of which are of a strong mixture modeling flavor. Motivated by this tradition, especially the work by von Davier (2009) and Hutchinson (1991), the current study presents a new mixture-modeling reformulation of the 3PLM by introducing an extra latent indicator for the guessing behavior and develops a feasible MLE algorithm, although this concept of mixture-modeling approach for the 3PLM has recurred in the IRT literature.

More specifically, a conceptual summary of developing such a new algorithm goes as: (a) introducing a new latent variable to construct a space one dimension higher than the old one, which appears to be unwise because the reformulation makes the original 3PLM estimation problem more difficult by adding one more dimension. (b) invoking the independent assumption of guessing and problem-solving process to approximate the joint distribution of the response and the newly introduced latent variable. It is worth noting that this practice is very common in statistics to approximate a high-dimension space. (c) using the approximation as a surrogate of the original 3PLM likelihood function to obtain item parameter estimate. Since the goal is to calibrate items with the traditional 3PLM, the convergence criterion is still calculated through the likelihood function of the original 3PLM, though the E and M steps are involved with the approximation of the likelihood function of the reformulated model.

The remaining is organized as the follows: Section 1 will present the reformulation of 3PLM based on the mixture modeling (McLachlan and Peel, 2004). In next section, EMM is developed and derivation of the estimation standard errors (SEs) for EMM is provided. In section 3, a comprehensive simulation study is conducted and the EMM is also applied to two empirical examples from BILOG-MG (Zimowski et al., 2003) and flexMIRT (Houts and Cai, 2015). The last section gives a brief discussion and future directions.

## 2. A mixture-modeling approach to 3PLM

Mixture modeling is a powerful statistical tool for representing the presence of heterogeneous subpopulations within an overall population. The generic density form of mixture modeling for a random vector *Y* can be written as

(1) $$f(y)=\sum_{i=1}^{g}\pi_{i}f_{i}(y),$$

in which the quantities π_1_, …, π_*g*_ are called the mixing proportions or weights for the *g* groups and the functions *f*_1_(*y*), …, *f*_*g*_(*y*) are called the component densities of the mixture. Obviously, mixture modeling overcomes the limitation of traditional modeling approach using one single density and attempts to approximate data by a linear combination of possibly various different densities. The idea of mixture modeling has been applied in psychometrics. Various Rasch mixture IRT models have been proposed to model different response styles and test taking strategies (Rost, 1997; von Davier and Rost, 2006). This paper takes advantage of this idea to reformulate 3PLM. If guessing was considered as a test-taking strategy, with introduction of a latent indicator variable for guessing, the 3PLM could be reformulated as a mixture model for two heterogeneous subpopulations: those who guess and those who do not, within each item. The detail derivation is presented below.

The 3PLM is formulated as the follow:

(2) $$P\left(u_{ij}=1|\theta_{j},a_{i},b_{i},c_{i}\right)\equiv P_{i}(\theta_{j})=c_{i}\times \left[1\right]+\left(1-c_{i}\right)\times \left[P_{i}^{*}(\theta_{j})\right],$$

with

(3) $$P_{i}^{*}\left(\theta_{j}\right)=\frac{1}{1+\text{exp}(-Da_{i}(\theta_{j}-b_{i}))}$$

in which *u*_*ij*_ ∈ **U** is examinee *j*(*j* = 1, 2, …, *N*)'s response to item *i*(*i* = 1, 2, …, *n*), *a*_*i*_, *b*_*i*_, and *c*_*i*_ are the item parameters; θ_*j*_ ∈ θ is the ability parameter of the examinee *j*; *D* is the scaling constant 1.702. The 3PLM can be considered as a mixture of a degenerate distribution (in which all the probability mass is on a single point) with a probability of *c*_*i*_ and a 2PLM with a probability of 1 − *c*_*i*_ within each item. Mixture models with a degenerate model is termed as nonstandard mixture models and has been studied intensively in statistics (Cornell University Library, 1989).

The observed data in a mixture problem is usually viewed as incomplete. A latent indicator variable *z*_*ij*_ ∈ **Z** for guessing is introduced:

$$z_{ij}=\{\begin{array}{c} \;1\text{ if examinee }j\text{ does not guess on item }i\text{;}\\ 0\text{ if examinee }j\text{ does guess on item }i\text{. } \end{array}$$

Thus, the marginal of *z*_*ij*_ follows a Bernoulli (1 − *c*_*i*_). And the conditional distribution of *u*_*ij*_ on *z*_*ij*_ is as the follow (let ξ_*i*_ = {*a*_*i*_, *b*_*i*_, *c*_*i*_} ∈ ξ represents the item parameter vector for the *i*th item):

(4) $$\begin{array}{l} P(u_{ij}=1|z_{ij}=1,\theta_{j},\xi_{i})=P_{i}^{*}(\theta_{j}),\;P(u_{ij}=1|z_{ij}=0,\theta_{j},\xi_{i})=1,\\ P(u_{ij}=0|z_{ij}=1,\theta_{j},\xi_{i})=1-P_{i}^{*}(\theta_{j}),\;P(u_{ij}=0|z_{ij}=0,\theta_{j},\xi_{i})=0. \end{array}$$

To simplify the derivation of the joint distribution of *u*_*ij*_ and *z*_*ij*_, *z*_*ij*_ and θ_*j*_ are assumed to be independent which leads to:

(5) $$P(u_{ij},z_{ij}|\theta_{j},\xi_{i})=P(u_{ij}|z_{ij},\theta_{j},\xi_{i})P(z_{ij}).$$

More specifically,

(6) $$\begin{array}{l} P(u_{ij}=1,z_{ij}=1|\theta_{j},\xi_{i})=\;(1-c_{i})P_{i}^{*}(\theta_{j}),\\ \;P(u_{ij}=1,z_{ij}=0|\theta_{j},\xi_{i})=c_{i},\\ P(u_{ij}=0,z_{ij}=1|\theta_{j},\xi_{i})=\;(1-c_{i})(1-P_{i}^{*}(\theta_{j})),\\ \;P(u_{ij}=0,z_{ij}=0|\theta_{j},\xi_{i})=0. \end{array}$$

Please note that the case of *u*_*ij*_ = 0 and *z*_*ij*_ = 0, whose probability is zero, is redundant, so the other three cases consist of the three elements of the joint distribution. The assumption of independence means that, for each item, the examinee decides randomly whether guessing or ability-based responding is chosen first (San Martín et al., 2006; von Davier, 2009). Let **u**_*j*_ and **z**_*j*_ be the response vector and the latent indicator vector for examinee *j*, respectively, and then the joint distribution for the new augmented complete data (**U**, **Z**, θ) is

(7) $$P(u_{j},z_{j},\theta_{j}|\xi ,\tau )=P(u_{j},z_{j}|\theta_{j},\xi )g(\theta_{j}|\tau ),$$

where

(8) $$\begin{array}{l} P\left(u_{j},z_{j}|\theta_{j},\xi \right)=\prod_{i=1}^{n}{\left[\left(1-c_{i}\right)P_{i}^{*}\left(\theta_{j}\right)\right]}^{u_{ij}z_{ij}}\times c_{i}^{u_{ij}\left(1-z_{ij}\right)}\\ \;\times \;{\left[\left(1-c_{i}\right)\left(1-P_{i}^{*}\left(\theta_{j}\right)\right)\right]}^{\left(1-u_{ij}\right)z_{ij}}, \end{array}$$

and *g*(θ_*j*_|τ) is a density function for θ, where τ is the vector containing the parameters of the examinee population ability distribution. Following Bock and Lieberman (1970), the marginal distribution for a single examinee *j* is:

(9) $$P(u_{j},z_{j}|\xi )=\int_{\theta_{j}}P(u_{j},z_{j}|\xi ,\theta_{j})g(\theta_{j}|\tau )d\theta_{j}.$$

So, the likelihood function of the EMM is:

(10) $$\begin{array}{l} L(U,Z|\xi )=\prod_{j=1}^{N}P(u_{j},z_{j}|\xi )\\ \;=\prod_{j=1}^{N}\int_{\theta_{j}}P(u_{j},z_{j}|\xi ,\theta_{j})g(\theta_{j}|\tau )d\theta_{j}. \end{array}$$

and the log-likelihood ln *L* = ln (*L*(**U**, **Z**|ξ)).

## 3. The expectation-maximization-maximization (EMM) algorithm

The reformulation of 3PLM points to the possibility that the guessing parameter can be estimated as a mixing parameter in mixture models, separated from the item discriminatory and difficulty parameters which will be estimated as the unknown parameters in the component density (the 2PLM). Put this in the language of the EM for IRT models and one has the Expectation (with respect to the latent variables Θ, **Z**)—Maximization (with respect to the guessing parameter (*c*_*i*_)—Maximization (with respect to the item discrimination and difficulty parameter *a*_*i*_ and *b*_*i*_). The first expectation step follows the same idea as the EM for IRT (Bock and Aitkin, 1981) with slight modification due to the introduction of the extra latent variable **Z**; the two maximization steps consist of the EM algorithm for the mixture models. In order to facilitate understanding, the description and mathematical notations of EMM in the sequel will follow Baker and Kim (2004). It is worthwhile noting that there is a tremendous similarity in the derivation of EMM and MMLE/EM with the only exception of the joint distribution of the complete data.

### 3.1. Expectation step and artificial data

Let ψ_*i*_ denote any item parameter for item *i* in ξ_*i*_, and very similar to the mathematical derivation for MMEL/EM in Baker and Kim (2004), the first derivative of the log-likelihood function in EMM for each item parameter can be obtained as (see Appendix A in Supplementary Material for details):

(11) $$\begin{array}{l} \frac{\partial \text{ ln }L}{\partial \psi_{i}}=\sum_{j=1}^{N}\frac{\partial \text{ ln }P(u_{j},z_{j}|\xi_{i})}{\partial \psi_{i}}\\ \;=\sum_{j=1}^{N}\frac{1}{P(u_{j},z_{j}|\xi_{i})}\frac{\partial P(u_{j},z_{j}|\xi_{i})}{\partial \psi_{i}}\\ \;=\sum_{j=1}^{N}\frac{1}{P(u_{j},z_{j}|\xi_{i})}\int_{\theta_{j}}\frac{\partial P(u_{j},z_{j}|\theta_{j},\xi_{i})g(\theta_{j}|\tau )d\theta_{j}}{\partial \psi_{i}}\\ \;=\sum_{j=1}^{N}\frac{1}{P(u_{j},z_{j}|\xi_{i})}\int_{\theta_{j}}\left[\frac{\partial \text{ ln }P(u_{j},z_{j}|\theta_{j},\xi_{i})}{\partial \psi_{i}}\right]\\ \;P(u_{j},z_{j}|\theta_{j},\xi_{i})g(\theta_{j}|\tau )d\theta_{j}\\ \;=\sum_{j=1}^{N}\int_{\theta_{j}}\left[\frac{\partial \text{ ln }P(u_{j},z_{j}|\theta_{j},\xi_{i})}{\partial \psi_{i}}\right]\left[\frac{P(u_{j},z_{j}|\theta_{j},\xi_{i})g(\theta_{j}|\tau )}{P(u_{j},z_{j}|\xi_{i})}\right]d\theta_{j}\\ \;=\sum_{j=1}^{N}\int_{\theta_{j}}\left[\frac{\partial \text{ ln }P(u_{j},z_{j}|\theta_{j},\xi_{i})}{\partial \psi_{i}}\right]P\left(\theta_{j}\;|\;u_{j},z_{j},\tau ,\xi_{i}\right)d\theta_{j}\\ \;=\sum_{j=1}^{N}\int_{\theta_{j}}[\frac{\partial }{\partial \psi_{i}}\text{ ln}\prod_{i=1}^{n}{\left[(1-c_{i})P_{i}^{*}(\theta_{j})\right]}^{u_{ij}z_{ij}}\times c_{i}^{u_{ij}(1-z_{ij})}\\ \;\times \;{\left[(1-c_{i})\left(1-P_{i}^{*}(\theta_{j})\right)\right]}^{(1-u_{ij})z_{ij}}]P\left(\theta_{j}\;u_{j},z_{j},\tau ,\xi \right)d\theta_{j}\\ \;=\sum_{j=1}^{N}\int_{\theta_{j}}[\frac{\left[u_{ij}-P_{i}^{*}(\theta_{j})\right]z_{ij}}{P_{i}^{*}(\theta_{j})\left[1-P_{i}^{*}(\theta_{j})\right]}\frac{\partial P_{i}^{*}(\theta_{j})}{\partial \psi_{i}}\\ \;+\;\frac{u_{ij}(1-z_{ij})}{c_{i}}\frac{\partial c_{i}}{\partial \psi_{i}}-\frac{z_{ij}}{1-c_{i}}\frac{\partial c_{i}}{\partial \psi_{i}}]P\left(\theta_{j}\;u_{j},z_{j},\tau ,\xi \right)d\theta_{j} \end{array}$$

with

(12) $$\begin{array}{l} P\left(\theta_{j}|u_{j},z_{j},\tau ,\xi \right)=P\left(\theta_{j}|u_{j},\tau ,\xi \right)=\frac{P(u_{j}|\theta_{j},\xi )g(\theta_{j}|\tau )}{\int_{\theta_{j}}P(u_{j}|\theta_{j},\xi )g(\theta_{j}|\tau )d\theta_{j}},\\ \;P(u_{j}|\theta_{j},\xi )=\prod_{i=1}^{n}P_{i}{\left(\theta_{j}\right)}^{u_{ij}}\times {\left(1-P_{i}(\theta_{j})\right)}^{1-u_{ij}}, \end{array}$$

where *P*(θ_*j*_|**u**_*j*_, **z**_*j*_, τ, ξ) is the posterior probability of θ_*j*_ given **u**_*j*_, **z**_*j*_, τ, andξ, and *P*(θ_*j*_|**u**_*j*_, **z**_*j*_, τ, ξ) always equals *P*(θ_*j*_|**u**_*j*_, τ, ξ) because of θ_*j*_ and **z**_*j*_ are assumed to be independent. The conditional expectation of *Z* is also essential for the E step. From the joint distribution in Equation (6), one can calculate the expectation of *z*_*ij*_ conditional on *u*_*ij*_ and the marginal distribution of *z*_*ij*_. By using the Bayesian rule, one has the conditional distribution of *z*_*ij*_ on *u*_*ij*_ as:

(13) $$\begin{array}{l} P(z_{ij}=1|u_{ij}=1,\theta_{j},\xi_{i})=\frac{\left(1-c_{i})P_{i}^{*}(\theta_{j}\right)}{P_{i}(\theta_{j})},\\ P(z_{ij}=1|u_{ij}=0,\theta_{j},\xi_{i})=1. \end{array}$$

Then the conditional expectation of *z*_*ij*_ is

(14) $$E(z_{ij}|u_{ij},\theta_{j},\xi_{i})=\frac{\left(1-c_{i})P_{i}^{*}(\theta_{j}\right)}{P_{i}(\theta_{j})}\times u_{ij}+1\times (1-u_{ij}).$$

The expectation step is to obtain the conditional expectation of the complete-data log likelihood with respect to the observation data, namely the response matrix **U**. In EMM it essentially amounts to plugging the conditional expectation of the indicator variable **Z** and integrating over the latent ability variable as in the original MMLE/EM. Let *X*_*k*_(*k* = 1, 2, …, *q*) be nodes on the ability scale with an associated weight *A*(*X*_*k*_), and the E step is as the follow:

(15) $$\begin{array}{l} \frac{\partial \text{ ln }E\left[L\right]}{\partial \psi_{i}}\\ \;\approx \sum_{j=1}^{N}\sum_{k=1}^{q}[\frac{\left[u_{ij}-P_{i}^{*}(\theta_{j})\right]z_{ij}}{P_{i}^{*}(\theta_{j})\left[1-P_{i}^{*}(\theta_{j})\right]}\frac{\partial P_{i}^{*}(\theta_{j})}{\partial \psi_{i}}+\frac{u_{ij}(1-E(z_{ij}|u_{ij},X_{k},\xi_{i}))}{c_{i}}\frac{\partial c_{i}}{\partial \psi_{i}}\\ \;-\frac{E(z_{ij}|u_{ij},X_{k},\xi_{i})}{1-c_{i}}\frac{\partial c_{i}}{\partial \psi_{i}}]P\left(X_{k}\;|u_{j},z_{j},\tau ,\xi \right) \end{array}$$

with

(16) $$\begin{array}{l} P\left(X_{k}|u_{j},z_{j},\tau ,\xi \right)=P\left(X_{k}|u_{j},\tau ,\xi \right)\\ \;=\frac{P(u_{j}|X_{k},\xi )A(X_{k})}{\sum_{k=1}^{q}P(u_{j}|X_{k},\xi )A(X_{k})},\\ P(u_{j}|X_{k},\xi )=\prod_{i=1}^{n}P_{i}{\left(X_{k}\right)}^{u_{ij}}\times {\left(1-P_{i}(X_{k})\right)}^{1-u_{ij}}, \end{array}$$

where *P*(*X*_*k*_ | **u**_*j*_, **z**_*j*_, τ, ξ) is the posterior probability of θ_*j*_ at *X*_*k*_ given **u**_*j*_, **z**_*j*_, τ, andξ, and *P*(*X*_*k*_| **u**_*j*_, **z**_*j*_, τ, ξ) always equals *P*(*X*_*k*_| **u**_*j*_, τ, ξ) because of the independence between *X*_*k*_| and **z**_*j*_. Furthermore, *P*(*X*_*k*_|**u**_*j*_, **z**_*j*_, τ, ξ) can be used to compute the “artificial data”. For instance, Bock and Aitkin (1981) has provided two fundamental artificial data for the traditional EM algorithm as:

(17) $$\begin{array}{l} {\bar{f}}_{ik}=\sum_{j=1}^{N}P\left(X_{k}|u_{j},\tau ,\xi \right)=\sum_{j=1}^{N}P\left(X_{k}|u_{j},z_{j},\tau ,\xi \right),\\ {\bar{r}}_{ik}=\sum_{j=1}^{N}u_{ij}\times P\left(X_{k}|u_{j},\tau ,\xi \right)=\sum_{j=1}^{N}u_{ij}\times P\left(X_{k}|u_{j},z_{j},\tau ,\xi \right), \end{array}$$

in which ${\overset{-}{f}}_{ik}$ is the expected number of examinees with ability *X*_*k*_. Thus, the sum of ${\overset{-}{f}}_{ik}$ for every ability *X*_*k*_ equals the total number of examinees *N*. The second index, ${\overset{-}{r}}_{ik}$, is the expected number of examinees with ability *X*_*k*_ answering item *i* correctly.

As can be seen from Table 1, the EMM algorithm introduced a new latent variable *Z*, so there are two new artificial data:

(18) $$\begin{array}{l} {\bar{f}}_{ik}^{\left(Z\right)}=\sum_{j=1}^{N}E(z_{ij}|u_{ij},X_{k},\xi )P\left(X_{k}|u_{j},z_{j},\tau ,\xi \right),\\ {\bar{r}}_{ik}^{\left(Z\right)}=\sum_{j=1}^{N}u_{ij}\times E(z_{ij}|u_{ij},X_{k},\xi )P\left(X_{k}|u_{j},z_{j},\tau ,\xi \right), \end{array}$$

in which ${\overset{-}{f}}_{ik}^{\left(\text{Z}\right)}$ is the expected number of examinees with ability *X*_*k*_ without using guessing strategy; ${\overset{-}{r}}_{ik}^{\left(\text{Z}\right)}$ is the expected number of examinees with ability *X*_*k*_ answering item *i* correctly without using the guessing strategy. Thus, ${\bar{r}}_{ik}-{\bar{r}}_{ik}^{\left(Z\right)}$ is the expected number of examinees with ability *X*_*k*_ who can answer item *i* correctly using the guessing strategy. The expected number of examinees with ability *X*_*k*_ who can answer item *i* incorrectly by using the guessing strategy is zero which can be inferred from *P*(*u*_*ij*_ = 0, *z*_*ij*_ = 0|θ_*j*_, ξ_*i*_) = 0. Putting these facts together, one can infer that ${\bar{r}}_{ik}-{\bar{r}}_{ik}^{\left(Z\right)}+{\bar{f}}_{ik}^{\left(Z\right)}$ across all is equivalent to the total number of examinees *N*, namely,

(19) $$\sum_{k=1}^{q}{\bar{f}}_{ik}=\sum_{k=1}^{q}\left({\bar{r}}_{ik}-{\bar{r}}_{ik}^{\left(Z\right)}+{\bar{f}}_{ik}^{\left(Z\right)}\right)=N.$$

After the E-step and calculation of the artificial data, the next steps are computing the first and second derivatives of Equation (15) with respect to each item parameter.

**Table 1:** The expected frequencies among examinees with ability *X*_*k*_ for item *i*.

**Item *i***	**z_*j*_ = 1**	**z_*j*_ = 0**	**Marginal of z_*j*_**
**u**_*j*_ = 1	${\overset{-}{r}}_{ik}^{\left(\text{z}\right)}$	${\overset{-}{r}}_{ik}-{\overset{-}{r}}_{ik}^{\left(\text{z}\right)}$	${\overset{-}{r}}_{ik}$
**u**_*j*_ = 0	${\overset{-}{f}}_{ik}^{\left(\text{z}\right)}-{\overset{-}{r}}_{ik}^{\left(\text{z}\right)}$	0	
Marginal of **u**_*j*_	${\overset{-}{f}}_{ik}^{\left(\text{z}\right)}$	${\overset{-}{r}}_{ik}-{\overset{-}{r}}_{ik}^{\left(\text{z}\right)}$	${\overset{-}{f}}_{ik}$

### 3.2. Maximization step-1 for the *c* parameter

Setting the first derivative equals to 0 and solving for *c*_*i*_:

(20) $$\begin{array}{l} 0=\lambda_{c_{i}}=\frac{\partial \text{ ln }E\left[L\right]}{\partial c_{i}}\\ \;\approx \sum_{j=1}^{N}\sum_{k=1}^{q}\left[\begin{array}{l} \frac{u_{ij}(1-E(z_{ij}|u_{ij},X_{k},\xi ))}{c_{i}}\\ -\frac{E(z_{ij}|u_{ij},X_{k},\xi )}{1-c_{i}} \end{array}\right]P\left(X_{k}\;|\;u_{j},z_{j},\tau ,\xi \right)\\ \;=\frac{\sum_{k=1}^{q}\left({\bar{r}}_{ik}-{\bar{r}}_{ik}^{\left(Z\right)}\right)}{c_{i}}-\frac{\sum_{k=1}^{q}{\bar{f}}_{ik}^{\left(Z\right)}}{1-c_{i}}, \end{array}$$

and surprisingly, a closed solution for *c*_*i*_ can be available as:

(21) $$\begin{array}{l} \Rightarrow \frac{\sum_{k=1}^{q}\left({\bar{r}}_{ik}-{\bar{r}}_{ik}^{\left(Z\right)}\right)}{c_{i}}=\frac{\sum_{k=1}^{q}{\bar{f}}_{ik}^{\left(Z\right)}}{1-c_{i}}\\ \Rightarrow c_{i}=\frac{\sum_{k=1}^{q}\left({\bar{r}}_{ik}-{\bar{r}}_{ik}^{\left(Z\right)}\right)}{\sum_{k=1}^{q}\left({\bar{f}}_{ik}^{\left(Z\right)}+{\bar{r}}_{ik}-{\bar{r}}_{ik}^{\left(Z\right)}\right)}=\frac{\sum_{k=1}^{q}\left({\bar{r}}_{ik}-{\bar{r}}_{ik}^{\left(Z\right)}\right)}{N}\\ \text{or }c_{i}=1-\frac{\sum_{k=1}^{q}{\bar{f}}_{ik}^{\left(Z\right)}}{N}. \end{array}$$

There is a nice interpretation for the estimate of the guessing parameter. From the description of the artificial data, it is easy to note that $\sum_{k=1}^{q}\left({\bar{r}}_{ik}-{\bar{r}}_{ik}^{\left(Z\right)}\right)$ is the expected number of examinees who can answer item *i* correctly using the guessing strategy and thus, the estimate is exactly the proportion of these examinees in the total sample. This interpretation presents a strong mixture modeling flavor, drastically different from the traditional interpretation, the lower bound for the probability with which a person solves the item correctly.

### 3.3. Maximization step-2 for *a* and *b* parameters

The second Maximization step is to execute the Newton-Raphson procedure to obtain estimates for *a*_*i*_ and *b*_*i*_. The required first derivatives for *a*_*i*_ and *b*_*i*_ are

(22) $$\begin{array}{l} \lambda_{a_{i}}=\frac{\partial \text{ ln }E\left[L\right]}{\partial a_{i}}=D\sum_{k=1}^{q}\left[({\bar{r}}_{ik}^{\left(Z\right)}-{\bar{f}}_{ik}^{\left(Z\right)}\times P_{i}^{*}(X_{k}))(X_{k}-b_{i})\right],\\ \lambda_{b_{i}}=\frac{\partial \text{ ln }E\left[L\right]}{\partial b_{i}}=-Da_{i}\sum_{k=1}^{q}[{\bar{r}}_{ik}^{\left(Z\right)}-{\bar{f}}_{ik}^{\left(Z\right)}\times P_{i}^{*}(X_{k})]. \end{array}$$

One may also set the derivatives to zero, but there is no analytical solution to them and thus Newton-Raphson method have to be used. In order to implement Newton-Raphson, the second derivatives are derived as

(23) $$\begin{array}{l} \lambda_{aa_{i}}=\frac{\partial^{2}\text{ ln }E\left[L\right]}{\partial a_{i}\partial a_{i}}=-D^{2}\sum_{k=1}^{q}{\left(X_{k}-b_{i}\right)}^{2}W_{ik}^{*}\times {\bar{f}}_{ik}^{\left(Z\right)},\\ \lambda_{bb_{i}}=\frac{\partial^{2}\text{ ln }E\left[L\right]}{\partial b_{i}\partial b_{i}}=-D^{2}a_{i}^{2}\sum_{k=1}^{q}W_{ik}^{*}\times {\bar{f}}_{ik}^{\left(Z\right)},\\ \lambda_{ab_{i}}=\frac{\partial^{2}\text{ ln }E\left[L\right]}{\partial a_{i}\partial b_{i}}=\sum_{k=1}^{q}\left[\begin{array}{l} D^{2}a_{i}(X_{k}-b_{i})W_{ik}^{*}\times {\bar{f}}_{ik}^{\left(Z\right)}\\ -D\left({\bar{r}}_{ik}^{\left(Z\right)}-{\bar{f}}_{ik}^{\left(Z\right)}\times P_{i}^{*}\left(\theta_{j}\right)\right) \end{array}\right], \end{array}$$

where

(24) $$W_{ik}^{*}=P_{i}^{*}(X_{k})\times (1-P_{i}^{*}(X_{k})),$$

So, the estimates can be obtained using the Newton-Raphson iteratively:

$$\left[\begin{array}{c} a_{i}^{\left(t+1\right)}\\ b_{i}^{\left(t+1\right)} \end{array}\right]=\left[\begin{array}{c} a_{i}^{\left(t\right)}\\ b_{i}^{\left(t\right)} \end{array}\right]-{\left[\begin{array}{cc} \lambda_{aa_{i}} & \lambda_{ab_{i}}\\ \lambda_{ab_{i}} & \lambda_{bb_{i}} \end{array}\right]}^{-1}\left[\begin{array}{c} \lambda_{a_{i}}\\ \lambda_{b_{i}} \end{array}\right].$$

### 3.4. Standard errors (SEs) of parameter estimation in EMM

One of the important indices to assess the estimation quality is the standard errors. One of the major criticisms of EM is, however, that parameter estimate SEs are not a natural product of the algorithm and some other methods have to be devised (McLachlan and Krishnan, 2007). EMM, as a member of EM algorithms, does not provide parameter estimate SEs either. In general, the inverse of the negative expected value of the matrix of second derivatives of a log likelihood is the asymptotic variance-covariance matrix of the estimates (McLachlan and Krishnan, 1968). The square roots of the diagonal elements of the inverse are the asymptotic standard errors of the parameters. This part will present a theoretical argument on why EMM works better than MMLE/EM for 3PLM. In the original MMLE/EM, the expected second derivative matrix can generically be written as

$$\left[\begin{array}{ccc} l_{aa} & l_{ab} & l_{ac}\\ l_{ab} & l_{bb} & l_{bc}\\ l_{ac} & l_{bc} & l_{cc} \end{array}\right],$$

in which *l*_*cc*_ is the expected second derivatives of the log likelihood with respect to the guessing parameter, *l*_*ac*_ and *l*_*bc*_ are the elements for two expected second partial derivatives. The convergence issue of the MMLE/EM for 3PLM is caused by this matrix being non-positive-definite or ill-conditioned (Baker and Kim, 2004). To solve this problem, several researchers suggested that the guessing parameter can usually set to be reciprocal of the number of the alternatives in the item (Hutchinson, 1991; Han, 2012; McDonald, 2013), which essentially mounted to set *l*_*ac*_ and *l*_*bc*_ to be zeros and then the 3PLM could be estimated. This option is available in several IRT programs, such as NOHARM (Fraser and Mcdonald, 1988), but an obvious limitation of this practice is that the guessing parameter is, in fact, not estimated, but a rather rough “guess” and does not seem to be a reasonable assumption (San Martín et al., 2006). In contrast, this matrix is different in EMM because EMM amounts to a divide-and-conquer strategy. The challenge of estimating item parameters is intelligently partitioned into two smaller estimation problems: estimation of the guessing parameter as the mixing proportion parameter in mixing modeling and that of the 2PL model. Interestingly, the two smaller problems happen to have been fully investigated in statistics and IRT. Conceptually, EMM can be perceived as a combination of these two mature techniques in statics and IRT. The prominent advantage of EMM comes from the separation of estimation of the guessing parameter from the other two and, thus, any instability in estimating the guessing parameter will not negatively affect that of the other two parameters, or otherwise. Such a setup statistically implies that the covariance between the guessing estimate and the other two are zeros. One may ensemble the expected second derivative matrix in generic form as

(25) $$\left[\begin{array}{ccc} E\left(-\lambda_{aa}|u_{ij}\right) & E\left(-\lambda_{ab}|u_{ij}\right) & 0\\ E\left(-\lambda_{ab}|u_{ij}\right) & E\left(-\lambda_{bb}|u_{ij}\right) & 0\\ 0 & 0 & E\left(-\lambda_{cc}|u_{ij}\right) \end{array}\right].$$

EMM provides a scientific alternative to the practice of fixing the guessing parameter estimate as the reciprocal of the number of the alternatives in the item. It can not only eliminate undesirable fluctuation in estimating the guessing parameter, but also make estimation of the guessing parameter possible. For the sake of completeness, the second derivatives of the log likelihood with respect to the guessing parameter is given as

(26) $$\lambda_{cc_{i}}=\frac{\partial^{2}\text{ ln }E\left[L\right]}{\partial^{2}c_{i}^{2}}=-\sum_{k=1}^{q}\left(\frac{{\bar{r}}_{ik}-{\bar{r}}_{ik}^{\left(Z\right)}}{c_{i}^{2}}+\frac{{\bar{f}}_{ik}^{\left(Z\right)}}{{\left(1-c_{i}\right)}^{2}}\right)$$

As a consequence of the separation setup, the calculation does not involve any terms related to the difficulty and discrimination parameters. The detailed derivation of the SEs for EMM is provided in Appendix B (Supplementary Material).

## 4. Simulation study

A simulation study was done to demonstrate the feasibility of the new method, compared to the Bayesian EM in BILOG-MG (Zimowski et al., 2003). The parameters for 10 or 20 items were generated from independent normal distribution as in Mislevy (1986): for ln *a*, the mean and variance were 0 and 0.5; for *b*, 0.5 and 1.0; for logit *c*, − 1.39 and 0.16.

**Figure 1:**
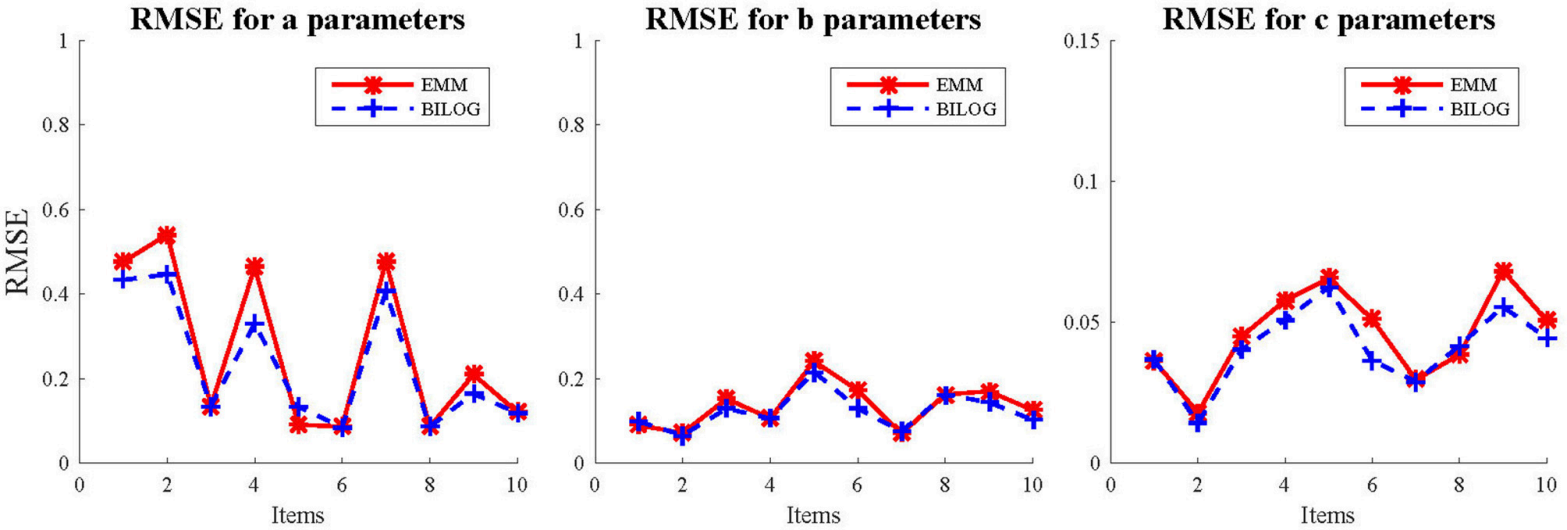
RMSE for item parameter estimates with 1,000 examinees and 10 items.

**Figure 2:**
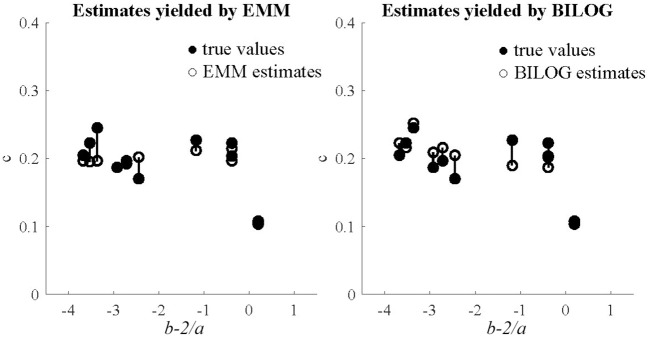
Generating and estimated values of *c*, against generating *b* − 2/*a* with 1,000 examinees and 10 items.

Three sample sizes of examinees (1,000, 1,500, and 2,000) were generated from standard normal distribution. 50 replications were run for each condition in the fully crossed 2(EMM vs. BILOG-MG) × 3(1, 000vs. 1, 500 vs. 2, 000) × 2(10vs. 20) design. The evaluation criteria are the bias and RMSE. In order to further demonstrate the performance of EMM, the EMM and Bayes solutions respectively, against generating values of the quantity *b* − 2/*a*, a heuristic index based on the observation that less information is obtained about *c* as items become easier or less reliable (Lord, 1975). Results. The detailed results for the simulation study are presented in tables and figures in Appendix C (Supplementary Material). Only the results for the condition of 1,000 examinees and 10 items are summarized and presented here (Figures 1, 2) since others are very similar. This is the most unfavorable condition for MLE since the sample size is moderately small and the test length is relatively short. But even under this condition, the EMM is comparable to or better than Bayesian EM in terms of bias and RMSE. In contrast, the traditional MMLE EM usually fails to converge with such a small sample size. As a MLE algorithm EMM is as flexible as the Bayesian EM implemented in BILOG-MG to deal with 3PL modeling in most practical testing situations.

The plots for the heuristic index comparing the EMM and Bayes solutions respectively, against generating values of the quantity *b* − 2/*a*, echo the results in terms of bias and RMSE. The figures for the 1,000-examinee-10-item condition are presented here. In general, both solutions for items with high values in the index are satisfactory, but the Bayes estimates for some items with low values were rather biased while the EMM estimates were very stable.

## 5. Two empirical examples

In order to further demonstrate the performance of the new algorithm, the authors apply EMM to two real data sets from BILOG-MG (Zimowski et al., 2003) and flexMIRT (Houts and Cai, 2015), and compare the item estimates to those from the two commercial programs. Specifically, the two data sets are the Example 1 in the BILOG-MG and Example 2 in flexMIRT. The BILOG-MG data set consists of 1,000 examinees' responses to 15 items and the one for flexMIRT consists of 2,844 examinees' responses to 12 items. Please refer to the manuals for the details. Both programs adopt different priors for the guessing parameter: BILOG-MG uses the default setting, a beta distribution with parameters of 4 and 16, namely, *c* ~ *Beta*(4, 16), and flexMIRT, *c* ~ *Beta*(1, 4). Please note that due to different parameterization in BILOG-MG technical document, the specification for the prior parameters are 5 and 17 which correspond to 4 and 16 in the standard beta distribution parameterization. The two priors have identical means, but the variance for flexMIRT prior is smaller which indicates that it is less informative. An additional analysis of flexMIRT data using the BILOG-MG default setting for the guessing parameter in flexMIRT [the *c* ~ *Beta*(1, 4) solution] is also run. In order to further facilitate the comparison of SEs, the authors employ supplemented EM (SEM) to obtain SEs for EMM (Meng and Rubin, 1991) in the real-data analysis below and SEM has been applied for various IRT models (Cai, 2008; Cai and Lee, 2009; Tian et al., 2013).

**Figure 3:**
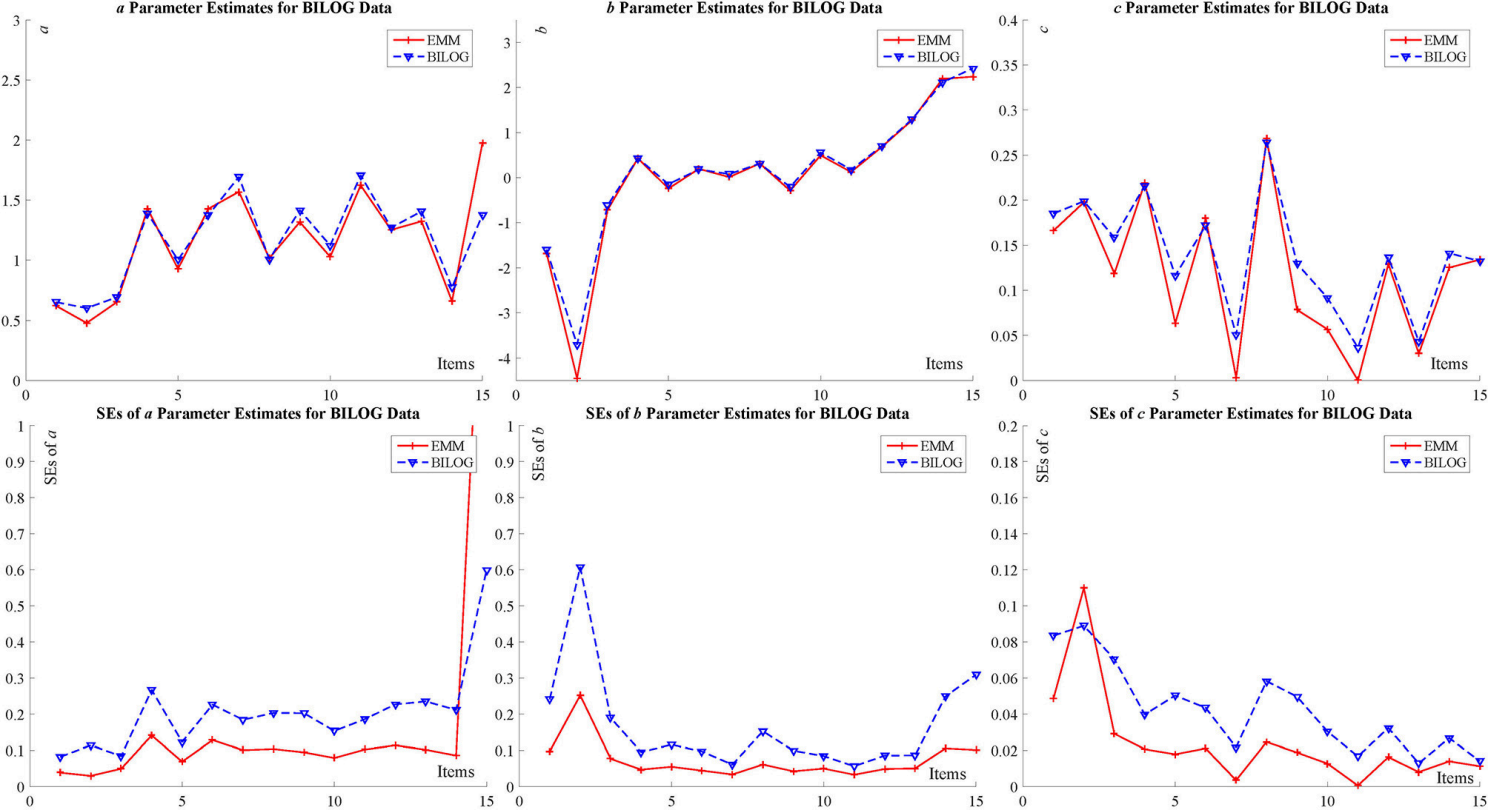
BILOG-MG data for item parameter estimate and SE with 1,000 examinees and 15 items.

**Figure 4:**
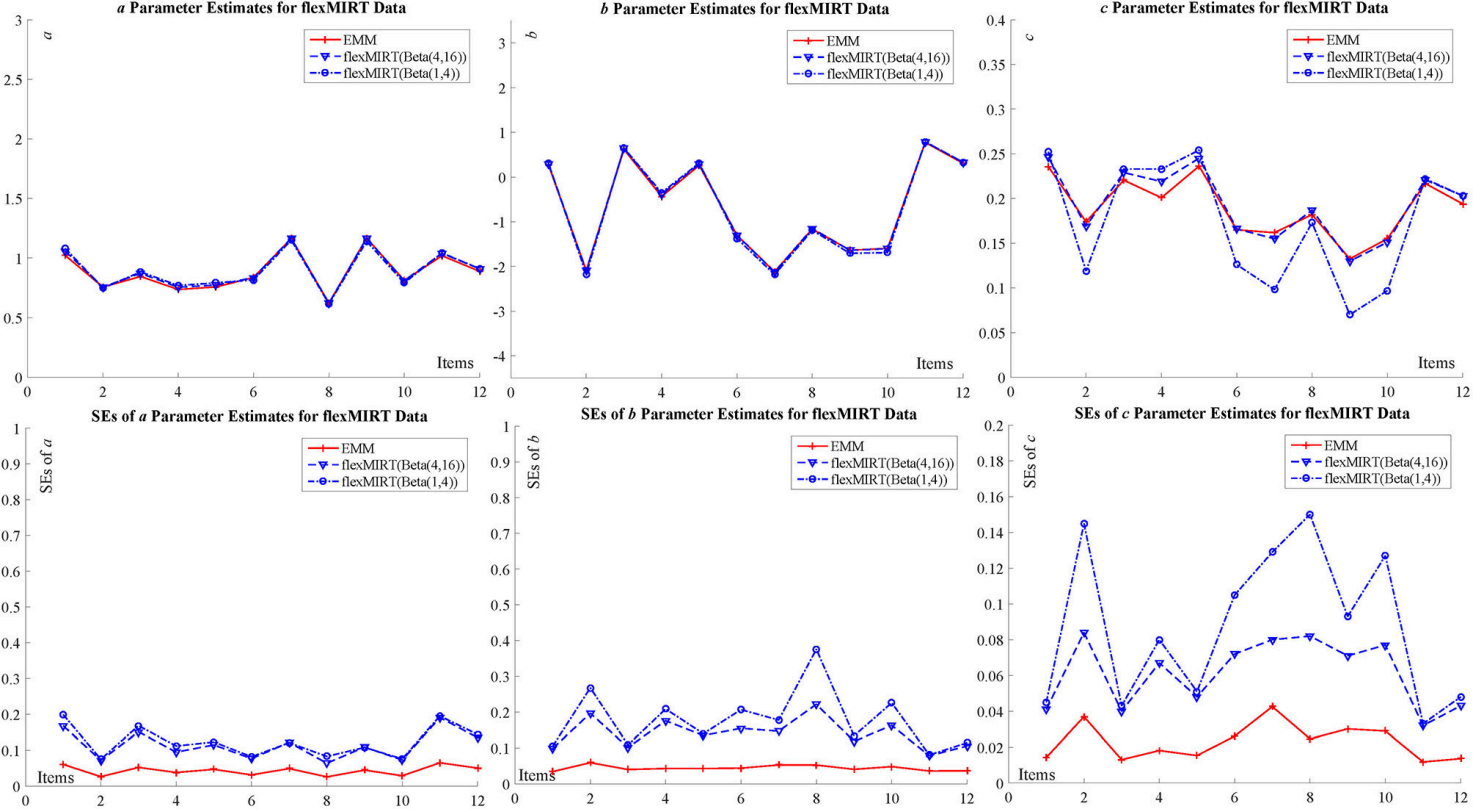
flexMIRT data for item parameter estimate and SE with 2,844 examinees and 12 items.

The specific results are summarized in the tables and figures in Appendix D (Supplementary Material) and only figures (Figure 3 for BILOG-MG data and Figure 4 for flexMIRT data) are presented here. Both Figures 3, 4 indicates that, for both real data sets, the point estimates (the upper part of the figures) for item parameters from EMM are comparable to those from BILOG-MG and flexMIRT, but SEs (the lower part of the figures) are smaller. Smaller SEs means that one may ascertain point estimates with greater confidence.

The advantage of EMM can be further confirmed by the difference between the *c* ~ *Beta*(1, 4) solution and the *c* ~ *Beta*(4, 16) solution to the point estimate for the guessing parameters for flexMIRT data. The EMM point estimates of the guessing parameters coincide with the *c* ~ *Beta*(4, 16) solution, with smaller SEs while those from the *c* ~ *Beta*(4, 16) solution present nontrivial differences. The authors may reasonably conclude that the difference between the solutions from flexMIRT calibration since the only difference in the specification is the prior setting for the guessing parameter. This difference is not a surprise at all because assigning proper priors is a common burden shared by the Bayesian approach. In the case of 3PLM calibration, however, different priors exert an undesirable negative effect of the point estimation and SEs. To exclude the possible difference in software implementation in the two programs, the authors also replicated the same analysis in BILOG-MG and the results corroborate the claim. Furthermore, as expected, the SEs in the *c* ~ *Beta*(1, 4) solution are bigger than those in the *c* ~ *Beta*(4, 16) solution which is another piece of evidence that priors in Bayesian methods lead to different SE estimates.

## 6. Discussion and future directions

In summary, the EMM essentially is a member of EM family for 3PLM. The fundamental difference between the EMM and the original EM is that the old complete data setup (**U**, θ) is expanded into (**U**, θ, **Z**). This change enables the algorithm to explore the likelihood function curve more thoroughly. More specifically, the challenge of estimating item parameters is intelligently partitioned into two smaller estimation problems: estimation of the guessing parameter as the mixing proportion parameter in mixing modeling and that of the 2PL model, which happen to be fully investigated in statistics and IRT. The benefit of this strategy is that the estimation error in the two problems does not exert any negative effect on each other and thus both of them are more stable. Moreover, for the guessing parameter, there is an analytical solution for the score function and thus the NR and the second derivatives are not necessary.

The EMM is a feasible algorithm to obtain MLE for 3PLM with modest sample size. It has several theoretical and practical implications. First, the mixture modeling approach to 3PLM is an interesting novel perspective on 3PLM. Second, this new method provides a feasible alternative for practitioners, enabling them to obtain MLE for 3PLM with modest sample size. This paper is not to advocate to eliminate use of Bayesian estimation methods. This can be used to check with the Bayesian solution with other IRT programs in practice. Due to the high complexity in real-world 3PLM data, a combination of EMM and Bayesian methods might lead to a more sophisticated and nuanced understanding of data.

Another important feature of EMM is that the EMM stopping criterion serves as a self-checking/correcting mechanism for the independence assumption. The stopping criterion is calculated through the original 3PLM likelihood function, although the E and M steps are built with the approximation of the likelihood function of the reformulated model. That is to say, the EMM would not converge until the difference between the values of the original 3PLM likelihood function evaluated at the current and last-cycle item estimates becomes negligible. If the independence assumption is severely violated and thus the approximation is not accurate enough, the convergence of the algorithm will suffer. In this sense, the EMM algorithm has provided a data-driven validation method for this assumption.

Several research questions deserve further attention. Firstly, there are two different interpretation of 3PLM (Hutchinson, 1991; San Martín et al., 2006; von Davier, 2009) and this paper chose one of them. The derivation of an EMM for the other interpretation will be very similar to this paper, but it needs to provoke different assumptions, so it would be interesting to compare the performance of two versions in terms of estimate and interpretation for the guessing parameter, numerical stability, etc. Secondly, a Bayesian EMM can be investigated. EMM is a more powerful MMLE method comparable to Bayesian EM, but the simulation studies indicate that there might be some inflation in item estimates in EMM which points to the possibility of improvement. A natural alternative is to combining the Bayesian approach and EMM. Bayesian EMM will solve the issue of estimate inflation while taking advantage of the EMM exploring the likelihood function. Thirdly, the mixture modeling approach and EMM can be naturally extended to 4PLM (Barton and Lord, 1981), which is a generalization of 3PLM and includes an upper asymptote for the probability of a correct response. There is a renewed interest in 4PLM (Rulison and Loken, 2009; Loken and Rulison, 2010; Liao et al., 2012; Ogasawara, 2012; Feuerstahler and Waller, 2014; Culpepper, 2016) for its usefulness in measuring psychological constructs. But, just as in the case of 3PLM, one consistent discussion point regarding 4PL pertains to the difficulty in estimating item parameters. The method proposed in this paper is a promising way for 4PLM.

## Author contributions

CZ: Conceptualization, Formal analysis, Investigation, Methodology, Project administration, Resources, Software, Supervision, Writing - original draft, Writing - review, and editing. XM: Funding acquisition and Software. SG: Software, Writing - review, and editing. ZL: Data curation, Project Administration, and Validation.

### Conflict of interest statement

The authors declare that the research was conducted in the absence of any commercial or financial relationships that could be construed as a potential conflict of interest.
