# Supplementary material for: Expectation-Maximization-Maximization: A Feasible MLE Algorithm for the Three-Parameter Logistic Model Based on a Mixture Modeling Reformulation
Source: Front Psychol. 2018 Jan 5;8:2302. doi: 10.3389/fpsyg.2017.02302 (PMC5760556; doi:10.3389/fpsyg.2017.02302)
Supplement: Supplementary file 1 [file Table1.PDF]

Supplementary Material: Expectation-Maximization-Maximization: A Feasible Mixture-model-based MLE Algorithm for the Three-parameter Logistic Model

1 APPENDIX A

This Appendix provides the full detail of the process in Equation (11).

$$
\begin{aligned}
0 = \frac{\partial \ln L}{\partial \psi_i} &= \sum_{j=1}^{N} \frac{\partial \ln P(\mathbf{u}_j, \mathbf{z}_j|\xi_i)}{\partial \psi_i} = \sum_{j=1}^{N} \frac{1}{P(\mathbf{u}_j, \mathbf{z}_j|\xi_i)} \frac{\partial P(\mathbf{u}_j, \mathbf{z}_j|\xi_i)}{\partial \psi_i} \\
&= \sum_{j=1}^{N} \frac{1}{P(\mathbf{u}_j, \mathbf{z}_j|\xi_i)} \int_{\theta_j} \frac{\partial P(\mathbf{u}_j, \mathbf{z}_j|\theta_j, \xi_i) g(\theta_j|\tau) d\theta_j}{\partial \psi_i} \\
&= \sum_{j=1}^{N} \frac{1}{P(\mathbf{u}_j, \mathbf{z}_j|\xi_i)} \int_{\theta_j} \left[\frac{\partial \ln P(\mathbf{u}_j, \mathbf{z}_j|\theta_j, \xi_i)}{\partial \psi_i}\right] P(\mathbf{u}_j, \mathbf{z}_j|\theta_j, \xi_i) g(\theta_j|\tau) d\theta_j \\
&= \sum_{j=1}^{N} \int_{\theta_j} \left[\frac{\partial \ln P(\mathbf{u}_j, \mathbf{z}_j|\theta_j, \xi_i)}{\partial \psi_i}\right] \left[\frac{P(\mathbf{u}_j, \mathbf{z}_j|\theta_j, \xi_i) g(\theta_j|\tau)}{P(\mathbf{u}_j, \mathbf{z}_j|\xi_i)}\right] d\theta_j \\
&= \sum_{j=1}^{N} \int_{\theta_j} \left[\frac{\partial \ln P(\mathbf{u}_j, \mathbf{z}_j|\theta_j, \xi_i)}{\partial \psi_i}\right] P\left(\theta_j|\mathbf{u}_j, \mathbf{z}_j, \tau, \xi_i\right) d\theta_j
\end{aligned}
$$

Let $P\left(\theta_j|\mathbf{u}_j, \mathbf{z}_j, \tau, \xi_i\right)$ equal $H(\theta_j)$, and then

$$
\begin{aligned}
&\Rightarrow \sum_{j=1}^{N} \int_{\theta_j} \left[\frac{\partial \ln P(\mathbf{u}_j, \mathbf{z}_j|\theta_j, \xi_i)}{\partial \psi_i}\right] H\left(\theta_j\right) d\theta_j \\
&= \sum_{j=1}^{N} \int_{\theta_j} \left[\frac{\partial}{\partial \psi_i} \ln \prod_{i=1}^{n} [(1-c_i) P_i^*(\theta_j)]^{u_{ij} z_{ij}} \times c_i^{u_{ij}(1-z_{ij})} \times [(1-c_i)\left(1-P_i^*(\theta_j)\right)]^{(1-u_{ij}) z_{ij}}\right] H\left(\theta_j\right) d\theta_j \\
&= \sum_{j=1}^{N} \int_{\theta_j} \left[\frac{\frac{\partial}{\partial \psi_i} \prod_{i=1}^{n} [(1-c_i) P_i^*(\theta_j)]^{u_{ij} z_{ij}} \times c_i^{u_{ij}(1-z_{ij})} \times [(1-c_i)\left(1-P_i^*(\theta_j)\right)]^{(1-u_{ij}) z_{ij}}}{\prod_{i=1}^{n} [(1-c_i) P_i^*(\theta_j)]^{u_{ij} z_{ij}} \times c_i^{u_{ij}(1-z_{ij})} \times [(1-c_i)\left(1-P_i^*(\theta_j)\right)]^{(1-u_{ij}) z_{ij}}}\right] H\left(\theta_j\right) d\theta_j
\end{aligned}
$$

$$= \sum_{j=1}^{N} \int_{\theta_j} \begin{bmatrix} [(1-c_i)P_i^*(\theta_j)]^{u_{ij}z_{ij}} \times c_i^{u_{ij}(1-z_{ij})} \times [(1-c_i)\left(1-P_i^*(\theta_j)\right)]^{(1-u_{ij})z_{ij}} \\ \times \prod_{h\neq i}^{n} [(1-c_h)P_h^*(\theta_j)]^{u_{hj}z_{hj}} \times c_h^{u_{hj}(1-z_{hj})} \times [(1-c_h)\left(1-P_h^*(\theta_j)\right)]^{(1-u_{hj})z_{hj}} \end{bmatrix}^{-1}$$

$$\times \frac{\partial}{\partial \psi_i} \begin{bmatrix} [(1-c_i)P_i^*(\theta_j)]^{u_{ij}z_{ij}} \times c_i^{u_{ij}(1-z_{ij})} \times [(1-c_i)\left(1-P_i^*(\theta_j)\right)]^{(1-u_{ij})z_{ij}} \\ \times \prod_{h\neq i}^{n} [(1-c_h)P_h^*(\theta_j)]^{u_{hj}z_{hj}} \times c_t^{u_{hj}(1-z_{hj})} \times [(1-c_h)\left(1-P_h^*(\theta_j)\right)]^{(1-u_{hj})z_{hj}} \end{bmatrix} H\left(\theta_j\right) d\theta_j$$

Let R_h=$[(1-c_h)P_h^*(\theta_j)]^{u_{hj}z_{hj}} \times c_h^{u_{hj}(1-z_{hj})} \times [(1-c_h)\left(1-P_h^*(\theta_j)\right)]^{(1-u_{hj})z_{hj}}$,

and R_i=$[(1-c_i)P_i^*(\theta_j)]^{u_{ij}z_{ij}} \times c_i^{u_{ij}(1-z_{ij})} \times [(1-c_i)\left(1-P_i^*(\theta_j)\right)]^{(1-u_{ij})z_{ij}}$, so we have

$$\text{RHS} = \sum_{j=1}^{N} \int_{\theta_j} \frac{\frac{\partial}{\partial \psi_i}\left[R_i \times \prod_{h\neq i}^{n} R_h\right]}{R_i \times \prod_{h\neq i}^{n} R_h} H\left(\theta_j\right) d\theta_j = \sum_{j=1}^{N} \int_{\theta_j} \frac{\left[\frac{\partial R_i}{\partial \psi_i}\right] \times \prod_{h\neq i}^{n} R_h + \left[R_i \times \frac{\partial}{\partial \psi_i} \prod_{h\neq i}^{n} R_h\right]}{R_i \times \prod_{h\neq i}^{n} R_h} H\left(\theta_j\right) d\theta_j$$

$$= \sum_{j=1}^{N} \int_{\theta_j} \left[\frac{1}{R_i \times \prod_{h\neq i}^{n} R_h} \left(\left[\frac{\partial R_i}{\partial \psi_i}\right] \times \prod_{h\neq i}^{n} R_h + R_i \times 0\right)\right] H\left(\theta_j\right) d\theta_j$$

$$= \sum_{j=1}^{N} \int_{\theta_j} \left[\frac{1}{R_i}\frac{\partial R_i}{\partial \psi_i}\right] H\left(\theta_j\right) d\theta_j$$

$$= \sum_{j=1}^{N} \int_{\theta_j} \left[\frac{1}{R_i} \frac{\partial[(1-c_i)P_i^*(\theta_j)]^{u_{ij}z_{ij}} \times c_i^{u_{ij}(1-z_{ij})} \times [(1-c_i)\left(1-P_i^*(\theta_j)\right)]^{(1-u_{ij})z_{ij}}}{\partial \psi_i}\right] H\left(\theta_j\right) d\theta_j$$

$$= \sum_{j=1}^{N} \int_{\theta_j} \frac{\begin{array}{l} \frac{\partial(1-c_i)P_i^*(\theta_j)}{\partial \psi_i} u_{ij}z_{ij}[(1-c_i)P_i^*(\theta_j)]^{u_{ij}z_{ij}-1} c_i^{u_{ij}(1-z_{ij})}[(1-c_i)\left(1-P_i^*(\theta_j)\right)]^{(1-u_{ij})z_{ij}} \\ + [(1-c_i)P_i^*(\theta_j)]^{u_{ij}z_{ij}} \frac{\partial c_i}{\partial \psi_i} u_{ij}(1-z_{ij}) c_i^{u_{ij}(1-z_{ij})-1}[(1-c_i)\left(1-P_i^*(\theta_j)\right)]^{(1-u_{ij})z_{ij}} \\ + [(1-c_i)P_i^*(\theta_j)]^{u_{ij}z_{ij}} \times c_i^{u_{ij}(1-z_{ij})} \times \frac{\partial(1-c_i)(1-P_i^*(\theta_j))}{\partial \psi_i} \\ \times (1-u_{ij})z_{ij} \times [(1-c_i)(1-P_i^*(\theta_j))]^{(1-u_{ij})z_{ij}-1} \end{array}}{R_i} H\left(\theta_j\right) d\theta_j$$

$$= \sum_{j=1}^{N} \int_{\theta_j} \left[\begin{array}{l} \dfrac{u_{ij} z_{ij}}{[(1-c_i)P_i^*(\theta_j)]} \left[P_i^*(\theta_j) \dfrac{\partial(1-c_i)}{\partial\psi_i} + (1-c_i) \dfrac{\partial P_i^*(\theta_j)}{\partial\psi_i} \right] + \dfrac{u_{ij}(1-z_{ij})}{c_i} \dfrac{\partial c_i}{\partial\psi_i} \\ + \dfrac{(1-u_{ij}) z_{ij}}{[(1-c_i)\left(1-P_i^*(\theta_j)\right)]} \left[(1-P_i^*(\theta_j)) \dfrac{\partial(1-c_i)}{\partial\psi_i} - (1-c_i) \dfrac{\partial P_i^*(\theta_j)}{\partial\psi_i} \right] \end{array} \right] H(\theta_j)\, d\theta_j$$

$$= \sum_{j=1}^{N} \int_{\theta_j} \left[\begin{array}{l} -\dfrac{u_{ij} z_{ij}}{1-c_i} \dfrac{\partial c_i}{\partial\psi_i} + \dfrac{u_{ij} z_{ij}}{P_i^*(\theta_j)} \dfrac{\partial P_i^*(\theta_j)}{\partial\psi_i} + \dfrac{u_{ij}(1-z_{ij})}{c_i} \dfrac{\partial c_i}{\partial\psi_i} \\ -\dfrac{(1-u_{ij}) z_{ij}}{1-c_i} \dfrac{\partial c_i}{\partial\psi_i} - \dfrac{(1-u_{ij}) z_{ij}}{1-P_i^*(\theta_j)} \dfrac{\partial P_i^*(\theta_j)}{\partial\psi_i} \end{array} \right] H(\theta_j)\, d\theta_j$$

$$= \sum_{j=1}^{N} \int_{\theta_j} \left[\begin{array}{l} -\left(\dfrac{u_{ij} z_{ij}}{1-c_i} + \dfrac{(1-u_{ij}) z_{ij}}{1-c_i} \right) \dfrac{\partial c_i}{\partial\psi_i} + \dfrac{u_{ij}(1-z_{ij})}{c_i} \dfrac{\partial c_i}{\partial\psi_i} \\ + \dfrac{u_{ij} z_{ij}}{P_i^*(\theta_j)} \dfrac{\partial P_i^*(\theta_j)}{\partial\psi_i} - \dfrac{(1-u_{ij}) z_{ij}}{1-P_i^*(\theta_j)} \dfrac{\partial P_i^*(\theta_j)}{\partial\psi_i} \end{array} \right] H(\theta_j)\, d\theta_j$$

$$= \sum_{j=1}^{N} \int_{\theta_j} \left[\begin{array}{l} \dfrac{u_{ij} z_{ij}}{P_i^*(\theta_j)} \dfrac{\partial P_i^*(\theta_j)}{\partial\psi_i} - \dfrac{(1-u_{ij}) z_{ij}}{1-P_i^*(\theta_j)} \dfrac{\partial P_i^*(\theta_j)}{\partial\psi_i} \\ + \dfrac{u_{ij}(1-z_{ij})}{c_i} \dfrac{\partial c_i}{\partial\psi_i} - \dfrac{z_{ij}}{1-c_i} \dfrac{\partial c_i}{\partial\psi_i} \end{array} \right] H(\theta_j)\, d\theta_j$$

$$= \sum_{j=1}^{N} \int_{\theta_j} \left[\begin{array}{l} \dfrac{u_{ij} z_{ij}}{[(1-c_i)P_i^*(\theta_j)]} \left[P_i^*(\theta_j) \dfrac{\partial(1-c_i)}{\partial\psi_i} + (1-c_i) \dfrac{\partial P_i^*(\theta_j)}{\partial\psi_i} \right] + \dfrac{u_{ij}(1-z_{ij})}{c_i} \dfrac{\partial c_i}{\partial\psi_i} \\ + \dfrac{(1-u_{ij}) z_{ij}}{[(1-c_i)\left(1-P_i^*(\theta_j)\right)]} \left[(1-P_i^*(\theta_j)) \dfrac{\partial(1-c_i)}{\partial\psi_i} - (1-c_i) \dfrac{\partial P_i^*(\theta_j)}{\partial\psi_i} \right] \end{array} \right] H(\theta_j)\, d\theta_j$$

$$= \sum_{j=1}^{N} \int_{\theta_j} \left[\begin{array}{l} -\dfrac{u_{ij} z_{ij}}{1-c_i} \dfrac{\partial c_i}{\partial\psi_i} + \dfrac{u_{ij} z_{ij}}{P_i^*(\theta_j)} \dfrac{\partial P_i^*(\theta_j)}{\partial\psi_i} + \dfrac{u_{ij}(1-z_{ij})}{c_i} \dfrac{\partial c_i}{\partial\psi_i} \\ -\dfrac{(1-u_{ij}) z_{ij}}{1-c_i} \dfrac{\partial c_i}{\partial\psi_i} - \dfrac{(1-u_{ij}) z_{ij}}{1-P_i^*(\theta_j)} \dfrac{\partial P_i^*(\theta_j)}{\partial\psi_i} \end{array} \right] H(\theta_j)\, d\theta_j$$

$$= \sum_{j=1}^{N} \int_{\theta_j} \left[\begin{array}{l} -\left(\dfrac{u_{ij} z_{ij}}{1-c_i} + \dfrac{(1-u_{ij}) z_{ij}}{1-c_i} \right) \dfrac{\partial c_i}{\partial\psi_i} + \dfrac{u_{ij}(1-z_{ij})}{c_i} \dfrac{\partial c_i}{\partial\psi_i} \\ + \dfrac{u_{ij} z_{ij}}{P_i^*(\theta_j)} \dfrac{\partial P_i^*(\theta_j)}{\partial\psi_i} - \dfrac{(1-u_{ij}) z_{ij}}{1-P_i^*(\theta_j)} \dfrac{\partial P_i^*(\theta_j)}{\partial\psi_i} \end{array} \right] H(\theta_j)\, d\theta_j$$

$$= \sum_{j=1}^{N} \int_{\theta_j} \left[\dfrac{u_{ij} z_{ij}}{P_i^*(\theta_j)} \dfrac{\partial P_i^*(\theta_j)}{\partial\psi_i} - \dfrac{(1-u_{ij}) z_{ij}}{1-P_i^*(\theta_j)} \dfrac{\partial P_i^*(\theta_j)}{\partial\psi_i} + \dfrac{u_{ij}(1-z_{ij})}{c_i} \dfrac{\partial c_i}{\partial\psi_i} - \dfrac{z_{ij}}{1-c_i} \dfrac{\partial c_i}{\partial\psi_i} \right] H(\theta_j)\, d\theta_j$$

$$= \sum_{j=1}^{N} \int_{\theta_j} \left[\dfrac{\left(u_{ij} - P_i^*(\theta_j)\right) z_{ij}}{P_i^*(\theta_j)\left[1-P_i^*(\theta_j)\right]} \dfrac{\partial P_i^*(\theta_j)}{\partial\psi_i} + \dfrac{u_{ij}(1-z_{ij})}{c_i} \dfrac{\partial c_i}{\partial\psi_i} - \dfrac{z_{ij}}{1-c_i} \dfrac{\partial c_i}{\partial\psi_i} \right] H(\theta_j)\, d\theta_j$$

where

$$H(\theta_j) = P(\theta_j | \mathbf{u}_j, \mathbf{z}_j, \tau, \xi) = P(\theta_j | \mathbf{u}_j, \tau, \xi) = \frac{P(\mathbf{u}_j|\theta_j,\xi) g(\theta_j|\tau)}{\int_{\theta_j} P(\mathbf{u}_j|\theta_j,\xi) g(\theta_j|\tau) d\theta_j},$$

$$P(\mathbf{u}_j|\theta_j,\xi) = \prod_{i=1}^{n} P_i(\theta_j)^{u_{ij}} \times (1 - P_i(\theta_j))^{1-u_{ij}}.$$

2 APPENDIX B

This is a derivation of the SEs in EMM. We have to derive the expected values of the artificial data first. From the derivation of the indicator variable z_{ij}, we have $E(z_{ij}|u_{ij},\theta_j,\xi_i) = \frac{(1-c_i)P_i^*(\theta_j)}{P_i(\theta_j)} \times u_{ij} + 1 \times (1-u_{ij})$. In 3PLM, setting $u_{ij} \sim \ Bernoulli\left(P_i\left(\theta_j\right)\right)$, so we can obtain:

$$
\begin{aligned}
&E[u_{ij}] = P_i(\theta_j),\\
&var(u_{ij}) = P_i(\theta_j) \times [1 - P_i(\theta_j)],\\
&E[u_{ij}^2] = var(u_{ij}) + E(u_{ij})^2\\
&= P_i(\theta_j) \times [1 - P_i(\theta_j)] + P_i(\theta_j)^2\\
&= P_i(\theta_j).
\end{aligned}
$$

So, the marginal z_{ij} is:

$$
\begin{aligned}
&E[E(z_{ij}|u_{ij},\theta_j,\xi_i)|u_{ij}]\\
&= \frac{(1-c_i)P_i^*(\theta_j)}{P_i(\theta_j)} \times P_i(\theta_j) + 1 \times (1 - P_i(\theta_j))\\
&= (1-c_i)P_i^*(\theta_j) + 1 - [c_i + (1-c_i)P_i^*(\theta_j)]\\
&= 1 - c_i\\
&= P(z_{ij} = 1).
\end{aligned}
$$

Plug this into the artificial data, we can have the expectations.

$$
\begin{aligned}
&E[\bar{f}_{ik}|u_{ij}] = E[\sum\nolimits_{j=1}^{N} P\left(X_k|\mathbf{u}_j,\mathbf{z}_j,\tau,\xi\right)|u_{ij}]\\
&= \sum\nolimits_{j=1}^{N} P\left(X_k|\mathbf{u}_j,\mathbf{z}_j,\tau,\xi\right)\\
&= \bar{f}_{ik},\\
&E[\bar{r}_{ik}|u_{ij}] = E[\sum\nolimits_{j=1}^{N} u_{ij} P\left(X_k|\mathbf{u}_j,\mathbf{z}_j,\tau,\xi\right)|u_{ij}]\\
&= P_i(X_k)\sum\nolimits_{j=1}^{N} P\left(X_k|\mathbf{u}_j,\mathbf{z}_j,\tau,\xi\right)\\
&= P_i(X_k)\bar{f}_{ik},\\
&E[\bar{f}_{ik}^{(Z)}|u_{ij}] = E[\sum\nolimits_{j=1}^{N} E(z_{ij}|u_{ij},X_k,\xi) P\left(X_k|\mathbf{u}_j,\mathbf{z}_j,\tau,\xi\right)|u_{ij}]\\
&= (1-c_i)\sum\nolimits_{j=1}^{N} P\left(X_k|\mathbf{u}_j,\mathbf{z}_j,\tau,\xi\right)\\
&= (1-c_i)\bar{f}_{ik},
\end{aligned}
$$

$$
\begin{aligned}
E[\bar{r}_{ik}^{(Z)}|u_{ij}] &= E[\sum\nolimits_{j=1}^{N} u_{ij}E(z_{ij}|u_{ij}, X_k, \xi)P\left(X_k|\mathbf{u}_j, \mathbf{z}_j, \tau, \xi\right)|u_{ij}] \\
&= E[\sum\nolimits_{j=1}^{N} (u_{ij}^2 \times \frac{(1-c_i)P_i^*(X_k)}{P_i(X_k)} + u_{ij} - u_{ij}^2)P\left(X_k|\mathbf{u}_j, \mathbf{z}_j, \tau, \xi\right)|u_{ij}] \\
&= \sum\nolimits_{j=1}^{N} (P_i(X_k) \times \frac{(1-c_i)P_i^*(X_k)}{P_i(X_k)} + P_i(X_k) - P_i(X_k))P\left(X_k|\mathbf{u}_j, \mathbf{z}_j, \tau, \xi\right) \\
&= (1-c_i)P_i^*(X_k)\sum\nolimits_{j=1}^{N} P\left(X_k|\mathbf{u}_j, \mathbf{z}_j, \tau, \xi\right) \\
&= P_i^*(X_k) \times (1-c_i)\bar{f}_{ik}.
\end{aligned}
$$

By now, the expected vales of the artificial data at X_k are re-expressed as the linear function of the expectation of $\bar{f}_{ik}$. $\bar{f}_{ik}$, which is of much help to simply the notation for the expectation of the second derivatives.

$$
\begin{aligned}
\gamma_{aa_i}^{EMM} &= E[\frac{\partial^2 \ln E\left[L\right]}{\partial^2 a_i^2}|u_{ij}] \\
&= E\left(-D^2 \sum_{k=1}^{q}\left[(X_k - b_i)^2 W_{ik}^* \times \bar{f}_{ik}^{(Z)}\right]\right) \\
&= -D^2 \sum_{k=1}^{q}\left[(X_k - b_i)^2 W_{ik}^* \times (1-c_i)\,\bar{f}_{ik}\right]
\end{aligned}
$$

where $W_{ik}^* = P_i^*\left(X_k\right)\left[1 - P_i^*\left(X_k\right)\right].$

$$
\begin{aligned}
\gamma_{bb_i}^{EMM} &= E[\frac{\partial^2 \ln E\left[L\right]}{\partial^2 b_i^2}|u_{ij}] = E\left(-D^2 a_i^2 \sum_{k=1}^{q}\left[\bar{f}_{ik}^{(Z)} \times W_{ik}^*\right]\right) \\
&= -D^2 a_i^2 \sum_{k=1}^{q}\left[(1-c_i)\,\bar{f}_{ik} \times W_{ik}^*\right]
\end{aligned}
$$

$$
\begin{aligned}
\gamma_{cc_i}^{EMM} &= E[\frac{\partial^2 \ln E\left[L\right]}{\partial^2 c_i^2}|u_{ij}] \\
&\approx E[\frac{\partial}{\partial c_i}\sum_{k=1}^{q}\left[\frac{\bar{r}_{ik} - \bar{r}_{ik}^{(Z)}}{c_i} - \frac{\bar{f}_{ik}^{(Z)}}{1-c_i}\right]|u_{ij}] \\
&= E[-\frac{\sum_{k=1}^{q}\bar{r}_{ik} - \bar{r}_{ik}^{(Z)}}{c_i^2} - \frac{\sum_{k=1}^{q}\bar{f}_{ik}^{(Z)}}{(1-c_i)^2}|u_{ij}] \\
&= -\frac{\sum_{k=1}^{q}\bar{f}_{ik} \times \left[p_i(X_k) - (1-c_i)p_i^*(X_k)\right]}{c_i^2} - \frac{\sum_{k=1}^{q}(1-c_i)\bar{f}_{ik}}{(1-c_i)^2} \\
&= -\frac{\sum_{k=1}^{q}\bar{f}_{ik}}{c_i} - \frac{\sum_{k=1}^{q}\bar{f}_{ik}}{(1-c_i)} = -\sum\nolimits_{k=1}^{q}\bar{f}_{ik}\left(\frac{(1-c_i)+c_i}{c_i(1-c_i)}\right) \\
&= -\frac{\sum_{k=1}^{q}\bar{f}_{ik}}{c_i(1-c_i)},
\end{aligned}
$$
