# Supplementary material for: Expectation-Maximization-Maximization: A Feasible MLE Algorithm for the Three-Parameter Logistic Model Based on a Mixture Modeling Reformulation
Source: Front Psychol. 2018 Jan 5;8:2302. doi: 10.3389/fpsyg.2017.02302 (PMC5760556; doi:10.3389/fpsyg.2017.02302)
Supplement: Supplementary file 2 [file Table2.DOCX]

Appendix C

| Table C1.  Bias for Item Parameter Estimates with 1000 Examinees and 10 Items | | | | | | | | | |
| --- | --- | --- | --- | --- | --- | --- | --- | --- | --- |
|  | Population value | | | *a* | | *b* | | *c* | |
| item | *a* | *b* | *c* | EMM | BILOG | EMM | BILOG | EMM | BILOG |
| 1 | 2.243 | 0.513 | 0.223 | -0.019 | -0.319 | -0.048 | -0.068 | -0.009 | -0.021 |
| 2 | 2.235 | 1.090 | 0.108 | -0.027 | -0.357 | -0.035 | -0.017 | -0.004 | -0.005 |
| 3 | 0.683 | -0.004 | 0.187 | 0.039 | 0.074 | -0.027 | 0.035 | 0.000 | 0.022 |
| 4 | 2.056 | -0.213 | 0.227 | 0.093 | -0.236 | -0.032 | -0.076 | -0.016 | -0.037 |
| 5 | 0.536 | 0.358 | 0.245 | -0.033 | 0.063 | -0.185 | 0.004 | -0.049 | 0.007 |
| 6 | 0.653 | -0.472 | 0.223 | -0.007 | 0.023 | -0.093 | -0.027 | -0.028 | -0.008 |
| 7 | 2.252 | 0.512 | 0.204 | 0.085 | -0.233 | -0.040 | -0.055 | -0.008 | -0.018 |
| 8 | 0.613 | -0.419 | 0.205 | -0.008 | 0.031 | -0.040 | 0.048 | -0.008 | 0.018 |
| 9 | 0.936 | -0.310 | 0.170 | 0.107 | 0.098 | 0.054 | 0.058 | 0.032 | 0.034 |
| 10 | 0.715 | 0.070 | 0.196 | 0.016 | 0.053 | -0.033 | 0.030 | -0.003 | 0.020 |

| Table C2.  Bias for Item Parameter Estimates with 1000 Examinees and 20 Items | | | | | | | | | |
| --- | --- | --- | --- | --- | --- | --- | --- | --- | --- |
|  | Population value | | | *a* | | *b* | | *c* | |
| item | *a* | *b* | *c* | EMM | BILOG | EMM | BILOG | EMM | BILOG |
| 1 | 2.318 | 0.990 | 0.268 | 0.148 | -0.224 | -0.032 | -0.040 | -0.003 | -0.012 |
| 2 | 0.436 | -0.491 | 0.179 | 0.018 | 0.068 | -0.022 | 0.167 | 0.004 | 0.049 |
| 3 | 1.353 | 0.829 | 0.199 | 0.174 | 0.103 | -0.046 | -0.044 | -0.003 | -0.005 |
| 4 | 1.860 | 0.438 | 0.321 | 0.129 | -0.122 | -0.047 | -0.085 | -0.006 | -0.028 |
| 5 | 2.189 | -1.645 | 0.182 | 0.112 | -0.339 | 0.015 | -0.066 | 0.010 | -0.002 |
| 6 | 1.252 | -0.386 | 0.233 | 0.039 | 0.007 | -0.029 | -0.041 | -0.016 | -0.022 |
| 7 | 0.601 | -0.266 | 0.159 | 0.036 | 0.075 | 0.012 | 0.111 | 0.011 | 0.046 |
| 8 | 2.159 | -1.761 | 0.190 | 0.233 | -0.291 | 0.022 | -0.074 | 0.004 | -0.009 |
| 9 | 1.400 | 0.657 | 0.130 | 0.126 | 0.114 | -0.029 | -0.017 | 0.007 | 0.012 |
| 10 | 0.455 | 1.229 | 0.139 | 0.084 | 0.189 | -0.007 | 0.084 | 0.027 | 0.073 |
| 11 | 0.571 | 1.264 | 0.241 | 0.014 | 0.130 | -0.138 | -0.044 | -0.025 | 0.019 |
| 12 | 0.785 | 0.364 | 0.259 | -0.012 | 0.012 | -0.125 | -0.083 | -0.035 | -0.021 |
| 13 | 0.483 | -0.370 | 0.266 | -0.027 | 0.025 | -0.266 | -0.073 | -0.069 | -0.024 |
| 14 | 2.186 | 0.861 | 0.090 | 0.261 | 0.125 | -0.035 | -0.026 | 0.003 | 0.006 |
| 15 | 1.317 | 0.876 | 0.160 | 0.152 | 0.114 | -0.043 | -0.035 | 0.005 | 0.006 |
| 16 | 0.744 | 0.862 | 0.131 | 0.085 | 0.143 | -0.026 | 0.026 | 0.006 | 0.030 |
| 17 | 0.585 | -0.540 | 0.211 | 0.024 | 0.058 | -0.052 | 0.043 | -0.019 | 0.010 |
| 18 | 0.784 | 0.605 | 0.234 | 0.041 | 0.064 | -0.079 | -0.051 | -0.012 | -0.002 |
| 19 | 2.493 | -0.313 | 0.180 | 0.018 | -0.308 | -0.039 | -0.056 | -0.003 | -0.010 |
| 20 | 1.933 | 1.539 | 0.233 | 0.518 | -0.055 | -0.108 | -0.088 | -0.002 | -0.009 |

| Table C3.  Bias for Item Parameter Estimates with 1500 Examinees and 10 Items | | | | | | | | | |
| --- | --- | --- | --- | --- | --- | --- | --- | --- | --- |
|  | Population value | | | *a* | | *b* | | *c* | |
| item | *a* | *b* | *c* | EMM | BILOG | EMM | BILOG | EMM | BILOG |
| 1 | 0.525 | 1.806 | 0.253 | -0.024 | 0.235 | -0.234 | -0.196 | -0.032 | 0.033 |
| 2 | 1.550 | 1.104 | 0.182 | 0.188 | 0.022 | -0.095 | -0.089 | 0.001 | -0.005 |
| 3 | 0.759 | 1.958 | 0.187 | 0.043 | 0.122 | -0.146 | -0.162 | -0.003 | 0.009 |
| 4 | 0.413 | 0.980 | 0.129 | 0.143 | 0.232 | 0.034 | 0.128 | 0.061 | 0.100 |
| 5 | 1.589 | 1.019 | 0.173 | 0.052 | -0.061 | -0.108 | -0.103 | -0.011 | -0.015 |
| 6 | 1.460 | -0.791 | 0.162 | 0.127 | 0.088 | -0.012 | -0.047 | 0.025 | 0.003 |
| 7 | 1.827 | 0.087 | 0.202 | -0.263 | -0.304 | -0.103 | -0.111 | -0.023 | -0.027 |
| 8 | 0.621 | 1.227 | 0.226 | 0.022 | 0.140 | -0.157 | -0.096 | -0.016 | 0.015 |
| 9 | 1.145 | 1.386 | 0.131 | 0.032 | 0.023 | -0.113 | -0.100 | 0.005 | 0.009 |
| 10 | 2.457 | 0.066 | 0.066 | -0.012 | -0.106 | -0.077 | -0.057 | -0.002 | 0.014 |

| Table C4.  Bias for Item Parameter Estimates with 1500 Examinees and 20 Items | | | | | | | | | |
| --- | --- | --- | --- | --- | --- | --- | --- | --- | --- |
|  | Population value | | | *a* | | *b* | | *c* | |
| item | *a* | *b* | *c* | EMM | BILOG | EMM | BILOG | EMM | BILOG |
| 1 | 1.322 | 0.679 | 0.167 | 0.032 | 0.027 | -0.038 | -0.027 | -0.009 | -0.006 |
| 2 | 0.509 | -0.064 | 0.262 | -0.040 | 0.017 | -0.256 | -0.053 | -0.070 | -0.016 |
| 3 | 2.000 | -0.967 | 0.249 | 0.039 | -0.131 | -0.010 | -0.040 | -0.031 | -0.045 |
| 4 | 0.848 | -0.754 | 0.180 | 0.034 | 0.050 | 0.016 | 0.054 | -0.002 | 0.016 |
| 5 | 1.966 | -0.206 | 0.281 | 0.123 | -0.071 | -0.013 | -0.043 | -0.009 | -0.028 |
| 6 | 1.197 | 0.439 | 0.184 | 0.066 | 0.062 | -0.033 | -0.023 | -0.005 | -0.002 |
| 7 | 1.662 | -1.720 | 0.139 | 0.179 | 0.055 | 0.107 | 0.075 | 0.053 | 0.048 |
| 8 | 0.681 | 1.783 | 0.246 | -0.059 | 0.110 | -0.092 | -0.097 | -0.036 | 0.000 |
| 9 | 2.209 | 0.863 | 0.147 | 0.132 | -0.075 | -0.035 | -0.028 | -0.002 | -0.004 |
| 10 | 0.475 | -1.446 | 0.189 | 0.007 | 0.033 | 0.003 | 0.127 | 0.003 | 0.029 |
| 11 | 0.393 | -1.273 | 0.201 | 0.016 | 0.057 | 0.117 | 0.195 | -0.004 | 0.026 |
| 12 | 0.469 | -0.936 | 0.209 | 0.005 | 0.047 | -0.048 | 0.129 | -0.015 | 0.028 |
| 13 | 0.365 | 0.418 | 0.091 | 0.059 | 0.143 | 0.251 | 0.439 | 0.066 | 0.126 |
| 14 | 1.308 | 1.515 | 0.245 | 0.002 | -0.064 | -0.040 | -0.031 | -0.010 | -0.012 |
| 15 | 0.338 | 0.533 | 0.245 | 0.001 | 0.117 | -0.341 | 0.095 | -0.063 | 0.036 |
| 16 | 0.347 | -0.269 | 0.186 | -0.006 | 0.055 | -0.121 | 0.226 | -0.011 | 0.052 |
| 17 | 1.030 | 0.589 | 0.294 | -0.008 | -0.016 | -0.064 | -0.059 | -0.021 | -0.022 |
| 18 | 0.906 | 0.540 | 0.154 | 0.035 | 0.070 | -0.020 | 0.015 | -0.007 | 0.008 |
| 19 | 1.738 | 0.580 | 0.240 | 0.106 | -0.015 | -0.033 | -0.040 | -0.001 | -0.008 |
| 20 | 1.638 | -0.328 | 0.310 | 0.006 | -0.091 | -0.039 | -0.068 | -0.035 | -0.052 |
| Table C5.  Bias for Item Parameter Estimates with 2000 Examinees and 10 Items | | | | | | | | | |
|  | Population value | | | *a* | | *b* | | *c* | |
| item | *a* | *b* | *c* | EMM | BILOG | EMM | BILOG | EMM | BILOG |
| 1 | 1.138 | 1.257 | 0.352 | -0.548 | -0.167 | -0.239 | -0.074 | -0.118 | -0.032 |
| 2 | 0.571 | -0.334 | 0.273 | -0.046 | 0.000 | -0.287 | -0.143 | -0.076 | -0.036 |
| 3 | 0.810 | -0.473 | 0.299 | -0.080 | -0.054 | -0.283 | -0.229 | -0.096 | -0.078 |
| 4 | 1.573 | 1.113 | 0.345 | -0.705 | -0.331 | -0.111 | -0.012 | -0.074 | -0.020 |
| 5 | 0.681 | 0.731 | 0.223 | -0.021 | 0.066 | -0.136 | -0.036 | -0.031 | 0.006 |
| 6 | 0.603 | -0.386 | 0.290 | -0.041 | -0.009 | -0.319 | -0.220 | -0.096 | -0.068 |
| 7 | 0.294 | 0.410 | 0.188 | -0.007 | 0.140 | -0.046 | 0.499 | -0.004 | 0.117 |
| 8 | 0.823 | -0.844 | 0.099 | 0.047 | 0.063 | 0.122 | 0.146 | 0.090 | 0.098 |
| 9 | 0.487 | -0.402 | 0.191 | 0.002 | 0.048 | -0.048 | 0.111 | 0.000 | 0.042 |
| 10 | 0.549 | 2.162 | 0.105 | 0.147 | 0.255 | -0.117 | -0.150 | 0.015 | 0.039 |

| Table C6.  Bias for Item Parameter Estimates with 2000 Examinees and 20 Items | | | | | | | | | |
| --- | --- | --- | --- | --- | --- | --- | --- | --- | --- |
|  | Population value | | | *a* | | *b* | | *c* | |
| item | *a* | *b* | *c* | EMM | BILOG | EMM | BILOG | EMM | BILOG |
| 1 | 1.418 | 0.856 | 0.228 | -0.040 | -0.121 | -0.061 | -0.070 | -0.015 | -0.023 |
| 2 | 1.946 | 0.515 | 0.264 | -0.017 | -0.211 | -0.061 | -0.093 | -0.017 | -0.033 |
| 3 | 0.716 | 2.232 | 0.124 | -0.021 | 0.050 | -0.007 | -0.022 | -0.012 | 0.003 |
| 4 | 1.044 | 0.911 | 0.182 | -0.017 | -0.031 | -0.060 | -0.048 | -0.016 | -0.015 |
| 5 | 2.187 | 1.830 | 0.133 | -0.045 | -0.316 | 0.015 | 0.062 | 0.000 | -0.001 |
| 6 | 2.344 | 0.473 | 0.215 | 0.038 | -0.230 | -0.045 | -0.072 | -0.005 | -0.020 |
| 7 | 2.110 | 1.776 | 0.135 | -0.003 | -0.265 | 0.008 | 0.052 | -0.001 | -0.002 |
| 8 | 1.833 | 1.500 | 0.266 | 0.036 | -0.188 | -0.016 | 0.003 | 0.001 | -0.005 |
| 9 | 1.177 | 0.714 | 0.210 | -0.015 | -0.055 | -0.058 | -0.063 | -0.015 | -0.019 |
| 10 | 0.478 | -0.423 | 0.232 | -0.007 | 0.020 | -0.170 | -0.072 | -0.046 | -0.023 |
| 11 | 0.433 | 0.995 | 0.273 | -0.077 | 0.027 | -0.399 | -0.091 | -0.098 | -0.018 |
| 12 | 2.010 | 0.728 | 0.115 | 0.051 | -0.067 | -0.031 | -0.029 | 0.000 | -0.002 |
| 13 | 1.153 | 1.115 | 0.171 | 0.024 | -0.014 | -0.029 | -0.019 | -0.002 | -0.003 |
| 14 | 1.353 | 2.516 | 0.116 | -0.066 | -0.113 | 0.139 | 0.118 | -0.010 | -0.002 |
| 15 | 2.237 | 1.557 | 0.171 | -0.135 | -0.379 | -0.010 | 0.018 | -0.002 | -0.005 |
| 16 | 0.785 | 0.517 | 0.271 | -0.091 | -0.041 | -0.208 | -0.129 | -0.067 | -0.041 |
| 17 | 2.116 | 1.869 | 0.274 | -0.165 | -0.389 | 0.047 | 0.067 | -0.003 | -0.003 |
| 18 | 1.524 | 2.053 | 0.224 | -0.076 | -0.185 | 0.018 | 0.059 | -0.004 | -0.004 |
| 19 | 0.688 | 2.609 | 0.091 | -0.043 | 0.059 | 0.049 | -0.035 | -0.010 | 0.006 |
| 20 | 1.369 | 1.212 | 0.203 | -0.002 | -0.088 | -0.027 | -0.021 | -0.001 | -0.006 |

| Table C7.  RMSE for Item Parameter Estimates with 1000 Examinees and 10 Items | | | | | | | | | |
| --- | --- | --- | --- | --- | --- | --- | --- | --- | --- |
|  | Population value | | | *a* | | *b* | | *c* | |
| item | *a* | *b* | *c* | EMM | BILOG | EMM | BILOG | EMM | BILOG |
| 1 | 2.243 | 0.513 | 0.223 | 0.476 | 0.434 | 0.089 | 0.098 | 0.036 | 0.037 |
| 2 | 2.235 | 1.090 | 0.108 | 0.540 | 0.446 | 0.071 | 0.065 | 0.018 | 0.014 |
| 3 | 0.683 | -0.004 | 0.187 | 0.135 | 0.135 | 0.152 | 0.129 | 0.045 | 0.040 |
| 4 | 2.056 | -0.213 | 0.227 | 0.463 | 0.330 | 0.106 | 0.106 | 0.058 | 0.051 |
| 5 | 0.536 | 0.358 | 0.245 | 0.090 | 0.133 | 0.241 | 0.216 | 0.065 | 0.062 |
| 6 | 0.653 | -0.472 | 0.223 | 0.086 | 0.084 | 0.172 | 0.129 | 0.051 | 0.036 |
| 7 | 2.252 | 0.512 | 0.204 | 0.477 | 0.406 | 0.070 | 0.075 | 0.030 | 0.029 |
| 8 | 0.613 | -0.419 | 0.205 | 0.085 | 0.086 | 0.162 | 0.161 | 0.038 | 0.042 |
| 9 | 0.936 | -0.310 | 0.170 | 0.209 | 0.164 | 0.170 | 0.143 | 0.068 | 0.055 |
| 10 | 0.715 | 0.070 | 0.196 | 0.121 | 0.118 | 0.125 | 0.102 | 0.051 | 0.044 |

| Table C8.  RMSE for Item Parameter Estimates with 1000 Examinees and 20 Items | | | | | | | | | |
| --- | --- | --- | --- | --- | --- | --- | --- | --- | --- |
|  | Population value | | | *a* | | *b* | | *c* | |
| item | *a* | *b* | *c* | EMM | BILOG | EMM | BILOG | EMM | BILOG |
| 1 | 2.318 | 0.990 | 0.268 | 0.506 | 0.439 | 0.069 | 0.074 | 0.025 | 0.027 |
| 2 | 0.436 | -0.491 | 0.179 | 0.078 | 0.101 | 0.194 | 0.241 | 0.041 | 0.063 |
| 3 | 1.353 | 0.829 | 0.199 | 0.373 | 0.273 | 0.091 | 0.083 | 0.035 | 0.028 |
| 4 | 1.860 | 0.438 | 0.321 | 0.392 | 0.286 | 0.100 | 0.119 | 0.041 | 0.047 |
| 5 | 2.189 | -1.645 | 0.182 | 0.459 | 0.429 | 0.086 | 0.093 | 0.033 | 0.016 |
| 6 | 1.252 | -0.386 | 0.233 | 0.216 | 0.168 | 0.116 | 0.094 | 0.058 | 0.043 |
| 7 | 0.601 | -0.266 | 0.159 | 0.101 | 0.112 | 0.191 | 0.189 | 0.060 | 0.063 |
| 8 | 2.159 | -1.761 | 0.190 | 0.556 | 0.460 | 0.094 | 0.123 | 0.026 | 0.015 |
| 9 | 1.400 | 0.657 | 0.130 | 0.258 | 0.215 | 0.073 | 0.063 | 0.025 | 0.022 |
| 10 | 0.455 | 1.229 | 0.139 | 0.170 | 0.227 | 0.218 | 0.206 | 0.063 | 0.083 |
| 11 | 0.571 | 1.264 | 0.241 | 0.199 | 0.207 | 0.243 | 0.188 | 0.061 | 0.047 |
| 12 | 0.785 | 0.364 | 0.259 | 0.144 | 0.115 | 0.217 | 0.159 | 0.071 | 0.048 |
| 13 | 0.483 | -0.370 | 0.266 | 0.067 | 0.072 | 0.322 | 0.210 | 0.079 | 0.052 |
| 14 | 2.186 | 0.861 | 0.090 | 0.427 | 0.321 | 0.056 | 0.050 | 0.015 | 0.014 |
| 15 | 1.317 | 0.876 | 0.160 | 0.315 | 0.246 | 0.082 | 0.075 | 0.030 | 0.024 |
| 16 | 0.744 | 0.862 | 0.131 | 0.185 | 0.195 | 0.134 | 0.114 | 0.046 | 0.042 |
| 17 | 0.585 | -0.540 | 0.211 | 0.091 | 0.097 | 0.177 | 0.143 | 0.060 | 0.044 |
| 18 | 0.784 | 0.605 | 0.234 | 0.141 | 0.123 | 0.212 | 0.169 | 0.066 | 0.049 |
| 19 | 2.493 | -0.313 | 0.180 | 0.454 | 0.416 | 0.085 | 0.079 | 0.052 | 0.037 |
| 20 | 1.933 | 1.539 | 0.233 | 0.783 | 0.343 | 0.131 | 0.119 | 0.016 | 0.018 |

| Table C9.  RMSE for Item Parameter Estimates with 1500 Examinees and 10 Items | | | | | | | | | |
| --- | --- | --- | --- | --- | --- | --- | --- | --- | --- |
|  | Population value | | | *a* | | *b* | | *c* | |
| item | *a* | *b* | *c* | EMM | BILOG | EMM | BILOG | EMM | BILOG |
| 1 | 0.525 | 1.806 | 0.253 | 0.103 | 0.274 | 0.307 | 0.260 | 0.051 | 0.052 |
| 2 | 1.550 | 1.104 | 0.182 | 0.384 | 0.235 | 0.106 | 0.101 | 0.017 | 0.015 |
| 3 | 0.759 | 1.958 | 0.187 | 0.137 | 0.164 | 0.165 | 0.179 | 0.031 | 0.024 |
| 4 | 0.413 | 0.980 | 0.129 | 0.205 | 0.277 | 0.119 | 0.197 | 0.074 | 0.105 |
| 5 | 1.589 | 1.019 | 0.173 | 0.244 | 0.184 | 0.118 | 0.114 | 0.020 | 0.020 |
| 6 | 1.460 | -0.791 | 0.162 | 0.249 | 0.208 | 0.061 | 0.067 | 0.033 | 0.015 |
| 7 | 1.827 | 0.087 | 0.202 | 0.317 | 0.336 | 0.106 | 0.115 | 0.032 | 0.032 |
| 8 | 0.621 | 1.227 | 0.226 | 0.128 | 0.235 | 0.207 | 0.151 | 0.065 | 0.061 |
| 9 | 1.145 | 1.386 | 0.131 | 0.292 | 0.195 | 0.126 | 0.113 | 0.026 | 0.021 |
| 10 | 2.457 | 0.066 | 0.066 | 0.357 | 0.227 | 0.091 | 0.065 | 0.026 | 0.020 |

| Table C10.  RMSE for Item Parameter Estimates with 1500 Examinees and 20 Items | | | | | | | | | |
| --- | --- | --- | --- | --- | --- | --- | --- | --- | --- |
|  | Population value | | | *a* | | *b* | | *c* | |
| item | *a* | *b* | *c* | EMM | BILOG | EMM | BILOG | EMM | BILOG |
| 1 | 1.322 | 0.679 | 0.167 | 0.197 | 0.167 | 0.076 | 0.064 | 0.032 | 0.026 |
| 2 | 0.509 | -0.064 | 0.262 | 0.064 | 0.072 | 0.286 | 0.181 | 0.077 | 0.051 |
| 3 | 2.000 | -0.967 | 0.249 | 0.373 | 0.255 | 0.096 | 0.083 | 0.064 | 0.059 |
| 4 | 0.848 | -0.754 | 0.180 | 0.095 | 0.098 | 0.107 | 0.107 | 0.046 | 0.041 |
| 5 | 1.966 | -0.206 | 0.281 | 0.436 | 0.325 | 0.086 | 0.086 | 0.048 | 0.048 |
| 6 | 1.197 | 0.439 | 0.184 | 0.201 | 0.174 | 0.084 | 0.071 | 0.030 | 0.024 |
| 7 | 1.662 | -1.720 | 0.139 | 0.352 | 0.227 | 0.136 | 0.107 | 0.059 | 0.051 |
| 8 | 0.681 | 1.783 | 0.246 | 0.189 | 0.200 | 0.205 | 0.168 | 0.053 | 0.028 |
| 9 | 2.209 | 0.863 | 0.147 | 0.432 | 0.306 | 0.048 | 0.043 | 0.016 | 0.015 |
| 10 | 0.475 | -1.446 | 0.189 | 0.057 | 0.064 | 0.209 | 0.221 | 0.019 | 0.037 |
| 11 | 0.393 | -1.273 | 0.201 | 0.073 | 0.085 | 0.767 | 0.311 | 0.060 | 0.053 |
| 12 | 0.469 | -0.936 | 0.209 | 0.063 | 0.095 | 0.203 | 0.289 | 0.036 | 0.065 |
| 13 | 0.365 | 0.418 | 0.091 | 0.086 | 0.160 | 0.301 | 0.479 | 0.073 | 0.131 |
| 14 | 1.308 | 1.515 | 0.245 | 0.298 | 0.218 | 0.100 | 0.098 | 0.026 | 0.023 |
| 15 | 0.338 | 0.533 | 0.245 | 0.077 | 0.169 | 0.377 | 0.350 | 0.073 | 0.081 |
| 16 | 0.347 | -0.269 | 0.186 | 0.063 | 0.087 | 0.355 | 0.332 | 0.025 | 0.070 |
| 17 | 1.030 | 0.589 | 0.294 | 0.200 | 0.160 | 0.137 | 0.121 | 0.052 | 0.044 |
| 18 | 0.906 | 0.540 | 0.154 | 0.127 | 0.124 | 0.116 | 0.096 | 0.045 | 0.035 |
| 19 | 1.738 | 0.580 | 0.240 | 0.333 | 0.239 | 0.062 | 0.064 | 0.024 | 0.024 |
| 20 | 1.638 | -0.328 | 0.310 | 0.252 | 0.203 | 0.127 | 0.122 | 0.072 | 0.074 |

| Table C11.  RMSE for Item Parameter Estimates with 2000 Examinees and 10 Items | | | | | | | | | |
| --- | --- | --- | --- | --- | --- | --- | --- | --- | --- |
|  | Population value | | | *a* | | *b* | | *c* | |
| item | *a* | *b* | *c* | EMM | BILOG | EMM | BILOG | EMM | BILOG |
| 1 | 1.138 | 1.257 | 0.352 | 0.574 | 0.325 | 0.274 | 0.163 | 0.124 | 0.052 |
| 2 | 0.571 | -0.334 | 0.273 | 0.072 | 0.072 | 0.306 | 0.224 | 0.078 | 0.057 |
| 3 | 0.810 | -0.473 | 0.299 | 0.119 | 0.103 | 0.298 | 0.250 | 0.097 | 0.083 |
| 4 | 1.573 | 1.113 | 0.345 | 0.756 | 0.424 | 0.147 | 0.099 | 0.082 | 0.030 |
| 5 | 0.681 | 0.731 | 0.223 | 0.124 | 0.166 | 0.168 | 0.118 | 0.047 | 0.042 |
| 6 | 0.603 | -0.386 | 0.290 | 0.087 | 0.081 | 0.339 | 0.251 | 0.097 | 0.073 |
| 7 | 0.294 | 0.410 | 0.188 | 0.048 | 0.196 | 0.140 | 0.584 | 0.012 | 0.134 |
| 8 | 0.823 | -0.844 | 0.099 | 0.125 | 0.127 | 0.143 | 0.163 | 0.093 | 0.102 |
| 9 | 0.487 | -0.402 | 0.191 | 0.070 | 0.085 | 0.115 | 0.184 | 0.018 | 0.056 |
| 10 | 0.549 | 2.162 | 0.105 | 0.274 | 0.310 | 0.284 | 0.247 | 0.033 | 0.045 |

| Table C12.  RMSE for Item Parameter Estimates with 2000 Examinees and 20 items | | | | | | | | | |
| --- | --- | --- | --- | --- | --- | --- | --- | --- | --- |
|  | Population value | | | *a* | | *b* | | *c* | |
| item | *a* | *b* | *c* | EMM | BILOG | EMM | BILOG | EMM | BILOG |
| 1 | 1.418 | 0.856 | 0.228 | 0.250 | 0.230 | 0.098 | 0.101 | 0.035 | 0.036 |
| 2 | 1.946 | 0.515 | 0.264 | 0.374 | 0.357 | 0.101 | 0.121 | 0.040 | 0.048 |
| 3 | 0.716 | 2.232 | 0.124 | 0.154 | 0.126 | 0.187 | 0.158 | 0.029 | 0.018 |
| 4 | 1.044 | 0.911 | 0.182 | 0.186 | 0.154 | 0.103 | 0.092 | 0.037 | 0.032 |
| 5 | 2.187 | 1.830 | 0.133 | 0.480 | 0.471 | 0.063 | 0.091 | 0.011 | 0.011 |
| 6 | 2.344 | 0.473 | 0.215 | 0.358 | 0.393 | 0.076 | 0.092 | 0.031 | 0.034 |
| 7 | 2.110 | 1.776 | 0.135 | 0.425 | 0.396 | 0.071 | 0.090 | 0.009 | 0.009 |
| 8 | 1.833 | 1.500 | 0.266 | 0.388 | 0.335 | 0.061 | 0.062 | 0.016 | 0.017 |
| 9 | 1.177 | 0.714 | 0.210 | 0.156 | 0.136 | 0.100 | 0.097 | 0.035 | 0.034 |
| 10 | 0.478 | -0.423 | 0.232 | 0.051 | 0.051 | 0.208 | 0.136 | 0.048 | 0.033 |
| 11 | 0.433 | 0.995 | 0.273 | 0.093 | 0.112 | 0.418 | 0.305 | 0.101 | 0.071 |
| 12 | 2.010 | 0.728 | 0.115 | 0.365 | 0.280 | 0.058 | 0.052 | 0.021 | 0.017 |
| 13 | 1.153 | 1.115 | 0.171 | 0.194 | 0.156 | 0.089 | 0.083 | 0.029 | 0.025 |
| 14 | 1.353 | 2.516 | 0.116 | 0.539 | 0.290 | 0.421 | 0.196 | 0.031 | 0.010 |
| 15 | 2.237 | 1.557 | 0.171 | 0.431 | 0.485 | 0.046 | 0.050 | 0.012 | 0.013 |
| 16 | 0.785 | 0.517 | 0.271 | 0.120 | 0.115 | 0.244 | 0.215 | 0.078 | 0.068 |
| 17 | 2.116 | 1.869 | 0.274 | 0.622 | 0.491 | 0.219 | 0.120 | 0.015 | 0.011 |
| 18 | 1.524 | 2.053 | 0.224 | 0.350 | 0.281 | 0.123 | 0.118 | 0.017 | 0.015 |
| 19 | 0.688 | 2.609 | 0.091 | 0.141 | 0.113 | 0.307 | 0.213 | 0.022 | 0.014 |
| 20 | 1.369 | 1.212 | 0.203 | 0.259 | 0.222 | 0.084 | 0.081 | 0.025 | 0.023 |

Figure C1.

Generating and EMM estimated values of c, against generating b-2/a

with 1000 examinees and 10 items

Figure C2.

Generating and BILOG estimated values of c, against generating b-2/a

with 1000 examinees and 10 items

Figure C3.

Generating and EMM estimated values of c, against generating b-2/a

with 1500 examinees and 10 items

Figure C4.

Generating and BILOG estimated values of c, against generating b-2/a

with 1500 examinees and 10 items

Figure C5.

Generating and EMM estimated values of c, against generating b-2/a

with 2000 examinees and 10 items

Figure C6.

Generating and BILOG estimated values of c, against generating b-2/a

with 2000 examinees and 10 items

Figure C7.

Generating and EMM estimated values of c, against generating b-2/a

with 1000 examinees and 20 items

Figure C8.

Generating and BILOG estimated values of c, against generating b-2/a

with 1000 examinees and 20 items

Figure C9.

Generating and EMM estimated values of c, against generating b-2/a

with 1500 examinees and 20 items

Figure C10.

Generating and BILOG estimated values of c, against generating b-2/a

with 1500 examinees and 20 items

Figure C11.

Generating and EMM estimated values of c, against generating b-2/a

with 2000 examinees and 20 items

Figure C12.

Generating and BILOG estimated values of c, against generating b-2/a

with 2000 examinees and 20 items

Figure C13.

RMSE for item parameter estimate with 1000 examinees and 10 items

Figure C14.

RMSE for item parameter estimate with 1000 examinees and 20 items

Figure C15.

RMSE for item parameter estimate with 1500 examinees and 10 items

Figure C16.

RMSE for item parameter estimate with 1500 examinees and 20 items

Figure C17.

RMSE for item parameter estimate with 2000 examinees and 10 items

Figure C18.

RMSE for item parameter estimate with 2000 examinees and 20 items

Appendix D

Table D1.

Item Parameter Estimates and SEs for BILOG-MG Data

| item | *a* | | *b* | | *c* | |
| --- | --- | --- | --- | --- | --- | --- |
|  | EMM | BILOG | EMM | BILOG | EMM | BILOG |
| 1 | 0.620 | 0.651 | -1.681 | -1.598 | 0.166 | 0.185 |
|  | (0.038) | (0.082) | (0.097) | (0.241) | (0.049) | (0.084) |
| 2 | 0.478 | 0.602 | -4.453 | -3.708 | 0.198 | 0.199 |
|  | (0.030) | (0.114) | (0.252) | (0.608) | (0.110) | (0.089) |
| 3 | 0.653 | 0.693 | -0.719 | -0.619 | 0.119 | 0.158 |
|  | (0.049) | (0.084) | (0.077) | (0.189) | (0.029) | (0.070) |
| 4 | 1.428 | 1.389 | 0.430 | 0.430 | 0.219 | 0.216 |
|  | (0.141) | (0.267) | (0.047) | (0.095) | (0.021) | (0.040) |
| 5 | 0.928 | 1.002 | -0.243 | -0.146 | 0.064 | 0.116 |
|  | (0.069) | (0.123) | (0.055) | (0.115) | (0.018) | (0.050) |
| 6 | 1.429 | 1.375 | 0.195 | 0.181 | 0.180 | 0.172 |
|  | (0.128) | (0.226) | (0.044) | (0.097) | (0.021) | (0.044) |
| 7 | 1.570 | 1.695 | 0.012 | 0.079 | 0.003 | 0.051 |
|  | (0.100) | (0.184) | (0.033) | (0.061) | (0.004) | (0.022) |
| 8 | 1.017 | 1.007 | 0.314 | 0.311 | 0.268 | 0.265 |
|  | (0.104) | (0.203) | (0.061) | (0.153) | (0.025) | (0.058) |
| 9 | 1.318 | 1.417 | -0.289 | -0.210 | 0.078 | 0.130 |
|  | (0.095) | (0.203) | (0.042) | (0.099) | (0.019) | (0.050) |
| 10 | 1.029 | 1.123 | 0.494 | 0.559 | 0.057 | 0.092 |
|  | (0.079) | (0.153) | (0.050) | (0.084) | (0.013) | (0.030) |
| 11 | 1.623 | 1.708 | 0.125 | 0.177 | 0.000 | 0.036 |
|  | (0.102) | (0.186) | (0.033) | (0.056) | (0.001) | (0.017) |
| 12 | 1.253 | 1.271 | 0.676 | 0.693 | 0.129 | 0.137 |
|  | (0.113) | (0.227) | (0.048) | (0.085) | (0.016) | (0.032) |
| 13 | 1.324 | 1.407 | 1.273 | 1.292 | 0.030 | 0.043 |
|  | (0.102) | (0.235) | (0.050) | (0.086) | (0.008) | (0.013) |
| 14 | 0.659 | 0.774 | 2.189 | 2.113 | 0.125 | 0.141 |
|  | (0.086) | (0.212) | (0.106) | (0.249) | (0.014) | (0.027) |
| 15 | 1.976 | 1.379 | 2.240 | 2.425 | 0.134 | 0.132 |
|  | (1.779) | (0.599) | (0.101) | (0.309) | (0.011) | (0.014) |

Table D2.

Item Parameter Estimates and SEs for flexMIRT Data

| item | *a* | | | *b* | | | *c* | | |
| --- | --- | --- | --- | --- | --- | --- | --- | --- | --- |
|  | EMM | flexMIRT | | EMM | flexMIRT | | EMM | flexMIRT | |
|  |  | *c~B*(4,16) | c~*B*(1,4) |  | *c~B*(4,16) | *c~B*(1,4) |  | *c~B*(4,16) | *c~B*(1,4) |
| 1 | 1.022 | 1.058 | 1.081 | 0.268 | 0.292 | 0.307 | 0.236 | 0.246 | 0.252 |
|  | (0.061) | (0.168) | (0.199) | (0.035) | (0.099) | (0.105) | (0.014) | (0.041) | (0.045) |
| 2 | 0.757 | 0.757 | 0.748 | -2.091 | -2.098 | -2.173 | 0.174 | 0.169 | 0.119 |
|  | (0.027) | (0.072) | (0.075) | (0.060) | (0.197) | (0.268) | (0.037) | (0.084) | (0.145) |
| 3 | 0.846 | 0.872 | 0.884 | 0.623 | 0.644 | 0.652 | 0.221 | 0.229 | 0.233 |
|  | (0.052) | (0.151) | (0.168) | (0.041) | (0.102) | (0.109) | (0.013) | (0.040) | (0.043) |
| 4 | 0.737 | 0.755 | 0.770 | -0.434 | -0.386 | -0.352 | 0.201 | 0.219 | 0.233 |
|  | (0.038) | (0.095) | (0.112) | (0.043) | (0.176) | (0.209) | (0.018) | (0.067) | (0.080) |
| 5 | 0.758 | 0.774 | 0.792 | 0.263 | 0.289 | 0.313 | 0.236 | 0.245 | 0.254 |
|  | (0.047) | (0.115) | (0.123) | (0.043) | (0.135) | (0.140) | (0.015) | (0.048) | (0.051) |
| 6 | 0.838 | 0.836 | 0.817 | -1.301 | -1.301 | -1.371 | 0.165 | 0.166 | 0.126 |
|  | (0.031) | (0.077) | (0.082) | (0.044) | (0.155) | (0.207) | (0.026) | (0.072) | (0.105) |
| 7 | 1.165 | 1.165 | 1.151 | -2.119 | -2.124 | -2.181 | 0.162 | 0.155 | 0.098 |
|  | (0.049) | (0.122) | (0.121) | (0.053) | (0.147) | (0.178) | (0.043) | (0.080) | (0.129) |
| 8 | 0.621 | 0.622 | 0.616 | -1.165 | -1.152 | -1.187 | 0.182 | 0.187 | 0.173 |
|  | (0.026) | (0.064) | (0.083) | (0.053) | (0.222) | (0.376) | (0.025) | (0.082) | (0.150) |
| 9 | 1.168 | 1.167 | 1.141 | -1.637 | -1.639 | -1.707 | 0.133 | 0.130 | 0.070 |
|  | (0.045) | (0.109) | (0.108) | (0.041) | (0.119) | (0.134) | (0.030) | (0.071) | (0.093) |
| 10 | 0.812 | 0.810 | 0.792 | -1.596 | -1.602 | -1.690 | 0.155 | 0.151 | 0.097 |
|  | (0.029) | (0.073) | (0.076) | (0.049) | (0.163) | (0.226) | (0.029) | (0.077) | (0.127) |
| 11 | 1.020 | 1.040 | 1.043 | 0.776 | 0.785 | 0.788 | 0.217 | 0.221 | 0.222 |
|  | (0.065) | (0.191) | (0.195) | (0.036) | (0.079) | (0.081) | (0.012) | (0.032) | (0.033) |
| 12 | 0.889 | 0.910 | 0.911 | 0.311 | 0.332 | 0.333 | 0.194 | 0.203 | 0.203 |
|  | (0.050) | (0.135) | (0.144) | (0.037) | (0.106) | (0.115) | (0.014) | (0.043) | (0.048) |

Figure D1.

BILOG data for item parameter estimate and SE with 1000 examinees and 15 items

Figure D2.

flexMIRT data for item parameter estimate and SE with 2844 examinees and 12 items
